# General Synthesis of 1-Aryl-6-azaisocytosines and Their Utilization for the Preparation of Related Condensed 1,2,4-Triazines

**DOI:** 10.3390/molecules24193558

**Published:** 2019-10-01

**Authors:** František Zálešák, Jan Slouka, Jakub Stýskala

**Affiliations:** Department of Organic Chemistry, Faculty of Science, Palacký University, 17. listopadu 1192/12, CZ-771 46 Olomouc, Czech Republic; frantisek.zalesak@upol.cz (F.Z.);

**Keywords:** 1-aryl-6-azaisocytosine, 1,2,4-triazine, condensed 1,2,4-triazine, fused azaisocytosines, cyclization of arylhydrazones

## Abstract

A simple general synthesis of 1-aryl-6-azaisocytosine-5-carbonitriles **4** is described. This method is based on coupling diazonium salts with cyanoacetylcyanamide **2** and then cyclization of the formed 2-arylhydrazono-2-cyanoacetylcyanamides **3**. The 6-azaisocytosines **4** were studied with respect to tautomeric equilibrium and the transformation of functional groups, and used in the synthesis of the condensed heterocyclic compounds: Purine isosteric imidazo[2,1-*c*]-[1,2,4]triazine **8** and the 1,2,4-triazino[2,3-*a*]quinazolines **9**–**12**.

## 1. Introduction

The 1,2,4-triazine ring is a prominent core structure that is found in numerous biologically active compounds, which have a wide range of potent pharmacological activities [1,2,3]. Certain aza analogs of pyrimidine nucleobases (6-azacytosine and 6-azauracil), which are structurally based on the 1,2,4-triazine heterocyclic system, have been shown to display antitumor, antiviral, antifungal, tuberculostatic, and anti-HIV activities.

The nucleosides of 6-azacytosine have shown antiproliferative activity against a wide variety of cancer cells. However, these azacytidines are deactivated in serum by the enzyme cytidine deaminase, resulting in inactive azauridines [4,5,6]. Hence, it is desirable to design and develop 6-azaisocytosine based molecules [6,7], which, as isomeric structures of 6-azacytosine, should be more resistant to enzymatic deamination [6]. In addition, fused azaisocytosines, where the nitrogen atom is part of a condensed ring, have promising medical applications as antiviral, antitumor, and antihemolytic agents [8].

The 6-aza analogs of pyrimidine nucleobases substituted with an aryl group at position 1 are of interest as 6-azacytosine analogs. For example, some 1-aryl-6-azauracils are used in veterinary medicine as antiprotozoal drugs (clazuril, diclazuril, nitromezuril) to combat the detrimental effects of protozoan parasites [9,10]. A substituted 1-aryl-6-azauracil-5-carbonitrile (MGL 3196) has been determined to be a highly selective thyroid hormone receptor β agonist, and is now in clinical trials for the treatment of dyslipidemia [11].

Unlike the 1-aryl-6-azauracils, which belong to the longest known and numerous derivatives of 1,2,4-triazine [12,13,14,15], there are only a few known derivatives of 1-aryl-6-azaisocytosines. These compounds can be prepared only with substitutions at the amino group at position 3 [16] or the nitrogen atom at position 4 [17] of the 1,2,4-triazine ring. Derivatives that are unsubstituted at these positions are not known and it is not possible to prepare them by established methods. To date, substituted 1-aryl-6-azaisocytosines have not been investigated in detail. These substances are interesting, not only in terms of potential biological activity, but also as useful starting materials for the synthesis of various condensed 1,2,4-triazines. In the preparation of potential new pharmaceutics, emphasis is placed on the use of readily available reactants using convenient and highly scalable reactions. Herein, we describe the development of a novel general method for the synthesis of 1-aryl-6-azaisocytosines (2-aryl-3-amino-1,2,4-triazin-5(2*H*)-ones).

**Figure 1:**
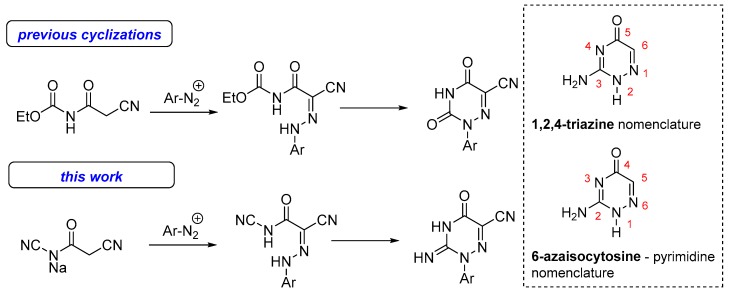
Schematic diagram of this work and atom numbering for the 6-azaisocytosine and 1,2,4-triazine skeletons.

For the preparation of 1-aryl-6-azaisocytosines, a previously known method [12,13,14,15] for the synthesis of 1-aryl-6-azauracils was modified using a cyanamide group instead of a carbamate (Figure 1). For subsequent modification of the newly prepared 1,2,4-triazines, we reacted the amino group in position 3 of the 1,2,4-triazine ring with functional groups located at position 2 of the attached aryl group, or with the nitrogen atom at position 4 of the 1,2,4-triazine ring. For example, using this method 1,2,4-triazino[2,3-*a*]quinazoline and purine isosteric imidazo[2,1-*c*][1,2,4]triazine derivatives could be obtained. These heterocycles are related to 6-azaisocytosines in which the nitrogen atom of amino group is incorporated into a fused ring.

## 2. Results and Discussion

**Scheme 1:**
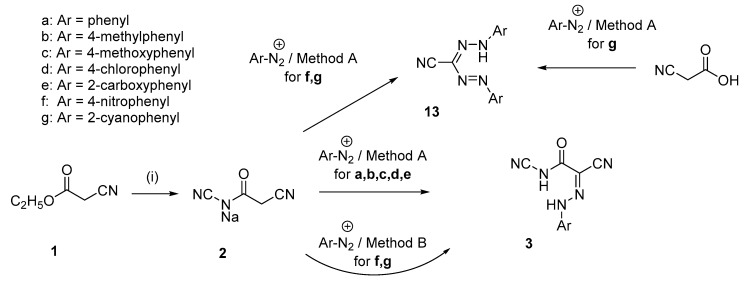
Preparation of arylhydrazones **3** showing the formation of undesired formazans **13** for very reactive diazonium salts **f, g.** *Reagents and conditions*: (i) NH_2_CN, EtONa, EtOH, reflux, 15 min; (Method A): Diazonium salt: ArNH_2_, HCl, H_2_O or AcOH (for **g**), NaNO_2_, 0–5 °C, 15–60 min, then addition to compound **2** in AcONa/H_2_O, 0–5 °C, 18 h; (Method B): Diazonium salt: Similar as above, then addition to compound **2** in H_2_O, 0–5 °C, 18 h.

2-Cyanoacetylcyanamide sodium salt **2** [18] was treated with diazonium salts to afford the corresponding hydrazones **3**. For the preparation of arylhydrazones **3a**–**3e** we used a general method for azo-coupling reactions using sodium acetate as a base (Method A). Unfortunately, this method failed with the benzenediazonium salts containing strong electron withdrawing groups (EWG) (NO_2_, CN). In this case, the formazans **13f**–**13g** were predominantly observed, as a mixture with the desired hydrazones **3f**–**3g**. The known cyano formazan **13g** [19] was also prepared for structure confirmation by different methods in this work based on the azo-coupling reaction of the 2-cyanobenzenediazonium salt with cyanoacetic acid. To avoid formazan **13** formation, we modified the azo-coupling process and carried out the reaction under strong acidic conditions without sodium acetate (Method B). This modified procedure enabled the preparation of the desired hydrazones **3f**–**3g** containing strong electron withdrawing groups in high yield. It is important to note that this method is not suitable for the preparation of the arylhydrazones **3a**–**3e** because of the insufficient electrophilicity of the corresponding diazonium salts (see Table 1 and Scheme 1). A similar coupling reaction in a strongly acidic environment has previously been performed using the reactive pyridine-1-oxide-2-diazonium salt [20].

**Scheme 2:**
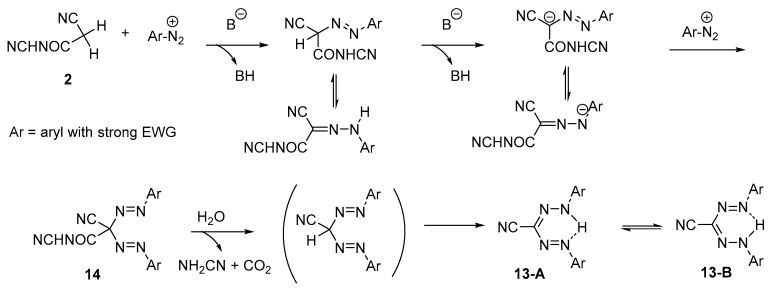
Plausible mechanisms for formazan **13** formation in the reaction of reactive diazonium salts with 2-cyanoacetylcyanamide **2** under basic conditions.

The different behaviors of the diazonium salts can be explained on the basis of their electrophilicity in the reaction with the twice-activated methylene group in cyanoacetylcyanamide **2**. It is well known that electrophilic agents can react with an activated methylene group. In the case of strong electrophiles, usually one group is sufficient for activation. For weaker electrophilic agents, such as diazonium salts, two activators are required. Such coupling reactions most often take place in basic medium forming azo compounds that are tautomeric with the corresponding hydrazones. However, when using reactive diazonium salts containing strong EWG in the reaction with cyanoacetylcyanamide **2** in basic medium, the azo compounds formed initially, deprotonated, and then a further azo-coupling reaction proceeded to give a double-coupled cyanoacetylcyanamide **14**, which formed a formazan after hydrolysis. In the case of reactive diazonium salts, this second azo coupling reaction leading to a formazan could be suppressed in a strongly acidic environment in which the mentioned subsequent azo-coupling reaction cannot take place (Scheme 2).

The prepared arylhydrazono-cyanoacetylcyanamides **3** are very poorly soluble in non-polar solvents. In polar solvents, particularly with traces of water, compounds **3** are transformed slowly to the less polar cyclized products **4** at room temperature. For this reason, the NMR data of arylhydrazones **3** were recorded immediately after sample preparation in DMSO-*d*_6_.

**Scheme 3:**
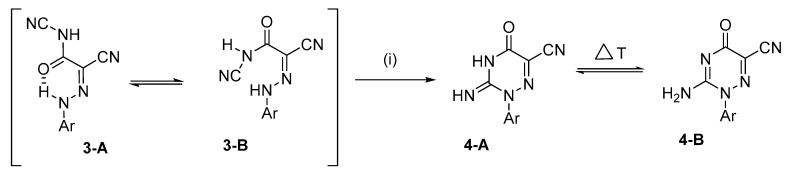
Cyclization of hydrazones **3** leading to 1-aryl-6-azaisocytosine-5-carbonitriles **4.** *Reagents and conditions*: (i) EtOH:H_2_O (3:1), reflux 60 min.

Compounds **3** (except **3e** and **3g**) were cyclized to the corresponding 1,2,4-triazines **4** by heating in a mixture of ethanol–water in good yields. It is interesting to note that in anhydrous ethanol this cyclization does not proceed (also not by adding of TsOH as an acid catalyst). The fact that hydrazones **3** do not cyclize on heating in anhydrous ethanol, but only on heating in aqueous ethanol, can be explained by the fact that these compounds **3** under normal conditions (or in anhydrous solvents) are present in the form **3-A**, which is stabilized by an intramolecular hydrogen bond and is unfavorable for the cyclization. This stable conformation prevents part of the molecule from spinning to form **3-B**, which is the conformation required to enable the addition of the NH group to the CN group. This intramolecular hydrogen bond is likely to be disrupted by the presence of water in polar solvents, or by heating the hydrazones **3** to a higher temperature, above the melting point, at which temperature the hydrogen bond also ceases to exist (see Scheme 3).

**Figure 2:**
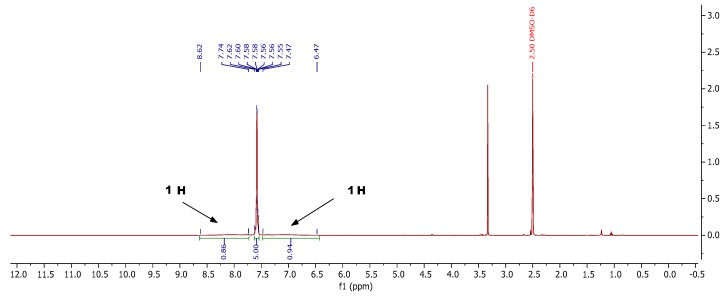
^1^H-NMR spectra of compound **4a** in DMSO-*d*_6_ recorded at 23 °C.

**Figure 3:**
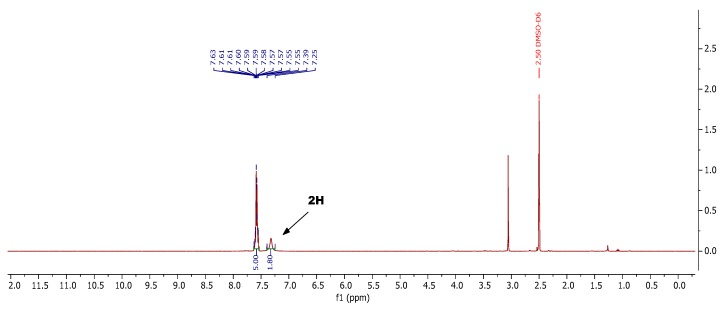
^1^H-NMR spectra of compound **4a** in DMSO-*d*_6_ recorded at 80 °C.

The prepared 1,2,4-triazines **4** (1-aryl-6-azaisocytosin-5-carbonitriles) can exist in two possible tautomeric forms **4-A** and **4-B**, which may influence further chemical modifications. To investigate these forms, the phenyl derivative **4a** was studied by ^1^H-NMR spectroscopy in DMSO-*d*_6_ at two different temperatures. We found that compound **4a** at laboratory temperature was present in the imino form **4-A** (two broad hydrogen atom signals were apparent at 8.2 and 7.0 ppm) (Figure 2), at a higher temperature of 80 °C, the amino tautomer **4-B** was observed (one hydrogen signal at 7.25 ppm with double the integral intensity) (Figure 3).

**Scheme 4:**
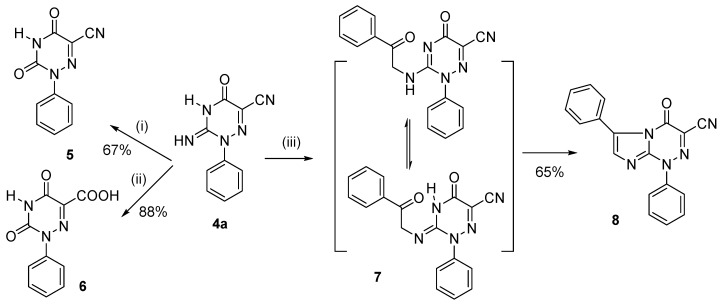
The hydrolytic reactions of 6-azaisocytosine **4a** and use in the synthesis of the condensed system **8**. *Reagents and conditions*. (i) 2% HCl in EtOH:H_2_O (1:1), reflux, 24 h; (ii) aq. 20% HCl, reflux, 2 h; (iii) PhCOCH_2_Br, DMF, 130 °C, 24 h.

The reactivity of the prepared 1-aryl-6-azaisocytosines **4**, with respect to the amino (imino) group in position 3 of the 1,2,4-triazine ring, was studied using the phenyl derivative **4a**. We found that the amino (imino) group underwent relatively easy hydrolysis under mild conditions to give the corresponding 1-phenyl-6-azauracil-5-carbonitrile **5 [12,15]** without hydrolysis of the nitrile group (Scheme 4). Under more drastic conditions [21], this hydrolysis resulted in the corresponding 1-phenyl-6-azauracil-5-carboxylic acid **6**.

The utility of the amino group of the 6-azaisocytosine skeleton for the synthesis of condensed 1,2,4-triazines was investigated. Formation of the fused ring between the amino group at position 3 and the nitrogen atom at position 4 of the 1,2,4-triazine cycle was demonstrated in the reaction of 1-phenyl-6-azaisocytosine-5-carbonitrile **4a** with phenacyl bromide, which resulted in the 2*N*-alkylated 6-azaisocytosine **7** which cyclized directly under the alkylation reaction conditions to the corresponding imidazo[2,1-*c*][1,2,4]triazine derivative **8**. There are two main possible centers for the alkylation of 1-phenyl-6-azaisocytosine **4a**: The amino group at position 3 and the nitrogen atom at position 4 of the 1,2,4-triazine cycle. The selective alkylation of compound **4a** at the amino group was accomplished at a high temperature and without a base, when only the amino form is present (proofed by NMR). It can be assumed that at 130 °C a thermodynamic equilibrium exists, in which the alkylation takes place at the more nucleophilic center—the amino group. In contrast, the nucleophilicity of the NH center at position 4 of the 1,2,4-triazine ring close to the C=O group is very low. Thus, the most probable cyclized product would be isomer **8** (Scheme 4).

**Scheme 5:**
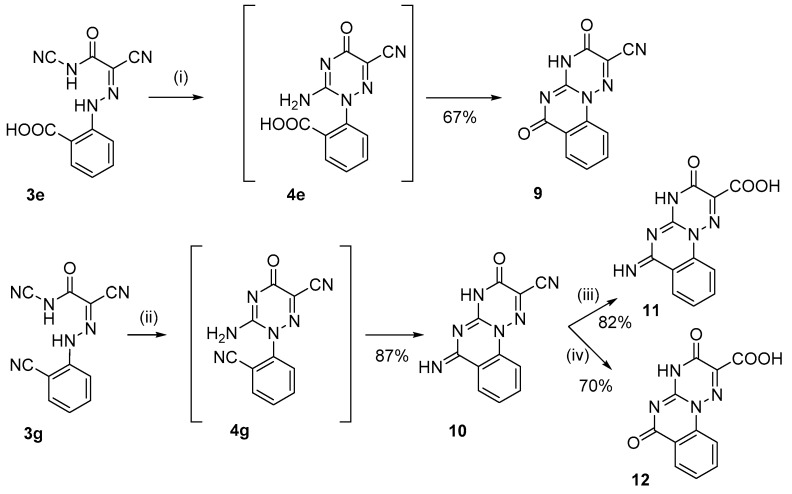
The preparation of 1,2,4-triazino[2,3-*a*]quinazoline derivatives **9**–**12**. *Reagents and conditions*. (i) EtOH:H_2_O (3:1), reflux, 24 h; (ii) EtOH:H_2_O (3:1), reflux, 60 min; (iii) aq. HCl (1:1), reflux, 20 min, then NH_3_, pH = 3–4; (iv) aq. 35% HCl, reflux, 2 h.

The reactivity of the amino (imino) group of compound **4a** with substituents located at position 2 of the attached aryl group was exploited for the preparation of 1,2,4-triazino[2,3-*a*]quinazoline derivatives. First, the cyclization reaction of (2-carboxyphenyl)hydrazone **3e**, formed the corresponding 1-(2-carboxyphenyl)-6-azaisocytosin-5-carbonitrile **4e**, which was used without isolation to smoothly provide 3,6-dioxo-3,4-dihydro-6*H*-triazino[2,3-*a*]quinazolin-2-carbonitrile **9** (Scheme 5). Thus, closure of the quinazoline ring by this lactamization was much more advantageous than closure of this ring by cyclocondensation of the amide group of 1-(*o*-carbamoylphenyl)-6-azauracil-5-carbonitrile [22]. A triazino-quinazoline skeleton was also prepared via a similar cyclocondensation of (2-cyanophenyl)hydrazone **3g**. In this reaction, the intermediate 1-(2-cyanophenyl)-6-azaisocytosin-5-carbonitrile **4g** was not observed and 6-imino-3-oxo-4,6-dihydro-3*H*-[1,2,4]triazino[2,3-*a*]quinazoline-2-carbonitrile **10** was prepared directly. The structure of compound **10** was unambiguously confirmed by IR spectroscopy in which only one CN group was apparent.

We were interested in the stability of the functional groups of compound **10** under acid hydrolysis. Surprisingly, we found the imino group to be more stable than the nitrile group. Under mild conditions, 6-imino-3-oxo-4,6-dihydro-3*H*-[1,2,4]triazino[2,3-*a*]quinazoline-2-carboxylic acid **11** was formed, whereas under more drastic conditions the hydrolysis resulted in formation of the 3,6-dioxo-4,6-dihydro-3*H*-[1,2,4]triazino[2,3-*a*]quinazoline-2-carboxylic acid **12.** We can conclude that the imino group in the quinazoline ring of compound **10** is much more stable to hydrolysis than the amino (imino) group connected to the 1,2,4-triazine ring of model compound **4a**.

**Figure 4:**
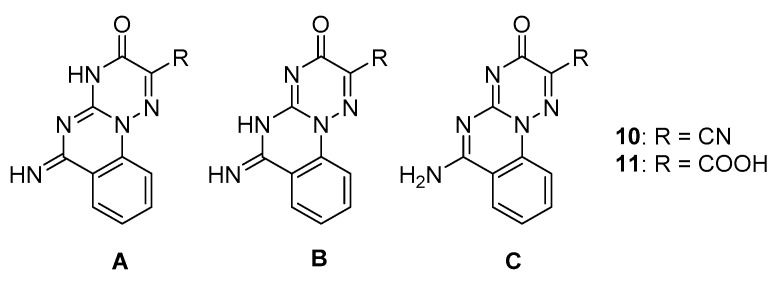
Possible tautomeric forms of 1,2,4-triazino[2,3-*a*]quinazolines **10** and **11**.

In addition to the given tautomer (**A**), compounds **10** and **11** may also be represented by two other tautomeric forms (**B**) and (**C**). In the varying tautomeric equilibrium between (**A**) and (**B**), two N-H groups are present; however, tautomer (**C**) has only an NH_2_ group (Figure 4). From the NMR data, it was apparent that the compounds did not contain an NH_2_ group (two sharp hydrogen signals were present in area of 9.0–9.3 ppm belonging to acidic N-H groups).

Many of the prepared compounds (especially condensed derivatives **9**–**12**) can serve as model compounds that have the potential for intercalation with DNA. These compounds may exhibit biological activity, not only through intercalation, as is the case with a number of isocyclic condensed aromatics [23,24,25,26], but also through forming intermolecular hydrogen bonds via the acidic NH groups.

## 3. Materials and Methods

### 3.1. General Informations

All commercially available reagents were used without further purification and purchased from standard chemical suppliers. Reactions were monitored by LC/MS analyses on a UHPLC-MS system (Thermo Scientific, Waltham, MA, USA) consisting of a UHPLC chromatograph equipped with a photodiode array detector and a triple quadrupole mass spectrometer using a C18 column at 30 °C and flow rate of 800 μL/min. Mobile phases: 10 mM ammonium acetate in HPLC grade water and HPLC grade acetonitrile. ^1^H- and ^13^C-NMR spectra were measured on an ECA 400II (^1^H: 399.78 MHz, ^13^C: 100.53 MHz,) NMR spectrometer (JEOL Resonance, Tokyo, Japan). Chemical shifts (δ) are reported in ppm and referenced to the middle peak of the solvent signal (DMSO-*d*_6_: 2.49 ppm, 39.50 ppm; CDCl_3_: 7.27 ppm, 77.00 ppm. All recorded ^1^H- and ^13^C-NMR spectra are available as Supplementary Materials online. NMR data of arylhydrazones **3** were recorded immediately after sample preparation in DMSO-*d*_6_. The IR spectra were recorded in KBr wafers on an ATI Unicam Genesis FTIR instrument. High resolution mass spectra (HRMS) measurements were performed on an Orbitrap Elite mass analyzer Thermo Exactive Plus equipped with Heated Electrospray Ionization (HESI) and Dionex Ultimate 3000 system (Thermo Scientific, MA, USA). Thin layer chromatography (TLC) was performed on pre-coated silica gel 60 F254 plates and visualized by exposure to UV light (254 or 366 nm). Melting points were measured on Boetius stage apparatus and are uncorrected.

### 3.2. Synthesis of Compounds ***2**–**13***

*2-Cyanoacetylcyanamide sodium salt* (**2**). To a solution of sodium ethoxide prepared from sodium (2.3 g, 0.1 mol) and anhydrous ethanol (50 mL) was added solution of cyanamide (4.2 g, 0.1 mol) in anhydrous ethanol (100 mL). After stirring at 50 °C for 5 min, to the formed suspension of sodium salt was added ethyl cyanoacetate (11.7 g, 0.1 mol). The resulting mixture was refluxed for 15 min and after cooling it was concentrated under reduced pressure to the volume of about 60 mL. This mixture was allowed to stand at 0–5 °C for 2 h. The precipitated white crystalline solid was filtered off, washed with cold ethanol and dried at 60 °C for 90 min. Yield was 11.7 g (85%). ^1^H-NMR (400 MHz, DMSO-*d*_6_): δ 3.27 (s, 2H); ^13^C-NMR (101 MHz, DMSO-*d*_6_): δ 27.3, 117.8, 120.7, 171.6.

#### 3.2.1. General Procedure for the Preparations of 2-Arylhydrazono-2-cyanoacetylcyanamides (**3**)

##### Method A

A solution of corresponding aniline (8 mmol) in a mixture of water (20 mL) and 35% hydrochloric acid (2 mL) was diazotized at 0–5 °C with a solution of sodium nitrite (0.55 g, 8 mmol) in ice water (10 mL). The mixture was stirred in ice bath for 15 min and then added portionwise to a solution of 2-cyanoacetylcyanamide sodium salt **2** (1.05 g, 8 mmol), sodium acetate (6 g) in water (50 mL), which was pre-cooled to 0–5 °C. After 20 min of stirring the reaction mixture was let to stand at 5 °C for 18 h. Then, aqueous solution of hydrochloric acid (1:2) was added to adjust pH to 3. The precipitated yellow-orange solid was filtered off, washed with water and dried.

##### Method B

Diazonium salt prepared in Method A was added with stirring to a solution of 2-cyanoacetylcyanamide sodium salt **2** (1.05 g, 8 mmol) in water (50 mL), which was pre-cooled to 0–5 °C. After 20 min of stirring the reaction mixture was let to stand at 5 °C for 18 h. Precipitated solid was filtered off, washed with water and dried.

*2-Phenylhydrazono-2-cyanoacetylcyanamide* (**3a**). This compound was prepared according to Method A using aniline hydrochloride (1.04 g). Yield 1.3 g (80%) orange solid, m.p. 166–170 °C. ^1^H-NMR (400 MHz, DMSO-*d*_6_): δ 7.17–7.21 (m, 1H, Ar), 7.39–7.44 (m, 2H, Ar), 7.72–7.75 (m, 2H, Ar), 12.06–12.78 (bs, 1H); ^13^C-NMR (101 MHz, DMSO-*d_6_*): δ 104.7, 108.5, 110.5, 116.8, 125.5, 129.3, 141.5, 161.9; IR (cm^−1^): 759, 1285, 1428, 1493, 1558, 1710, 2213 (CN), 2259 (CN), 3228; HRMS (HESI *m/z*): [M + H]^+^ calcd for C_10_H_8_N_5_O 214.0723; found 214.0724.

*2-(4-Methylphenylhydrazono)-2-cyanoacetylcyanamide* (**3b**). This compound was prepared according to Method A using 4-methylaniline (0.86 g). Yield 1.60 g (87%) yellow solid, m.p. 162–166 °C. ^1^H-NMR (400 MHz, DMSO-*d*_6_): δ 2.30 (s, 3H, CH_3_), 7.22 (d, *J* = 8.3 Hz, 2H, Ar) 7.63 (d, *J* = 8.4 Hz, 2H, Ar), 12.04–12.63 (bs, 1H); ^13^C-NMR (101 MHz, DMSO-*d*_6_): δ 20.5, 103.9, 108.5, 110.6, 116.8, 129.7, 134.9, 139.3, 161.9; IR (cm^−1^): 815, 1299, 1447, 1608, 1678, 2217 (CN), 2254 (CN), 3237; HRMS (HESI *m/z*): [M + H]^+^ calcd for C_11_H_10_N_5_O 228.0880; found 228.0881.

*2-(4-Methoxyphenylhydrazono)-2-cyanoacetylcyanamide* (**3c**). This compound was prepared according to Method A using 4-methoxyaniline (0.98 g). Yield 1.62 g (83%) yellow-orange solid, m.p. 166–170 °C (with observed cyclization). ^1^H-NMR (400 MHz, DMSO-*d*_6_): δ 3.77 (s, 3H, OCH_3_), 6.96–7.00 (m, 2H, Ar), 7.67–7.71 (m, 2H, Ar), 12.32–12.56 (bs, 1H); ^13^C-NMR (101 MHz, DMSO-*d*_6_): δ 55.4, 103.1, 108.6, 110.8, 114.5, 118.4, 135.1, 157.4, 162.0; IR (cm^−1^): 1033, 1253, 1447, 1562, 1691, 2217 (CN), 2254 (CN), 3205; HRMS (HESI *m/z*): [M + H]^+^ calcd for C_11_H_10_N_5_O_2_ 244.0829; found 244.029.

*2-(4-Chlorophenylhydrazono)-2-cyanoacetylcyanamide* (**3d**). This compound was prepared according to Method A using 4-chloroaniline (1.02 g). Yield 1.47 g (79%) yellow-orange solid, m.p. 166–168 °C (with cyclization). ^1^H-NMR (400 MHz, DMSO-*d*_6_): δ 7.45–7.49 (m, 2H, Ar), 7.72–7.76 (m, 2H,Ar), 12.06–12.87 (bs, 1H); ^13^C-NMR (101 MHz, DMSO-*d*_6_): δ 105.5, 108.6, 110.4, 118.4, 129.2, 129.3, 140.5, 161.9; IR (cm^−1^): 838, 1290, 1456, 1562, 1682,2218 (CN), 2264 (CN), 3089, 3242; HRMS (HESI *m/z*): [M + H]^+^ calcd for C_10_H_7_ClN_5_O 248.0334; found 248.0333.

*2-(2-Carboxyphenylhydrazono)-2-cyanoacetylcyanamide* (**3e**). This compound was prepared according to Method A using 2-aminobenzoic acid (1.1 g). Yield 1.55 g (72%) yellow solid, m.p. 198–202 °C. ^1^H-NMR (400 MHz, DMSO-*d*_6_): δ 7.09–7.13 (m, 1H, Ar), 7.60–7.64 (m, 1H, Ar), 7.73 (d, *J* = 8.3 Hz, 1H, Ar), 7.95 (dd, *J* = 7.9, 1.2 Hz, 1H, Ar), 12.41–12.86 (bs, 1H); ^13^C-NMR (101 MHz, DMSO-*d*_6_): δ 112.2, 113.9, 115.1, 122.5, 131.3, 134.8, 144.4, 166.4, 169.5; IR (cm^−1^): 755, 1253, 1530, 1604, 1673, 2176 (CN), 2250 (CN), 3491; HRMS (HESI *m/z*): [M − H]^−^ calcd for C_11_H_6_N_5_O_3_ 256.0476; found 256.0476.

*2-(4-Nitrophenylhydrazono)-2-cyanoacetylcyanamide* (**3f**). This compound was prepared according to Method B using 4-nitroaniline (1.1 g). Yield 1.91 g (93%) yellow-orange solid m.p. 185–186 °C; ^1^H-NMR (400 MHz, DMSO-*d_6_*): δ 7.87–7.91 (m, 2H, Ar), 8.26-8.29 (m, 2H, Ar); ^13^C-NMR (101 MHz, DMSO-*d*_6_): δ 110.1, 112.4, 116.7, 125.3, 126.4, 143.5, 161.9; IR (cm^−1^): 847, 1258, 1341, 1530, 1595, 2167 (CN), 2365 (CN), 3228, 3412; HRMS (HESI *m/z*): [M + H]^+^ calcd for C_10_H_7_N_6_O_3_ 259.0574; found 259.0572.

*2-(2-Cyanophenylhydrazono)-2-cyanoacetylcyanamide* (**3g**). This compound was prepared according to Method B with different diazotation process. A solution of 2-aminobenzencabonitrile (0.472 g, 4 mmol) in a mixture of acetic acid (10.5 mL) and 35% hydrochloric acid (3 mL) was diazotized at 0–5 °C with a solution of sodium nitrite (0.275 g, 4 mmol) in ice water (3 mL). The mixture was stirred in ice bath for 1 h and then added portionwise to a solution of 2-cyanoacetylcyanamide sodium salt **2** (0.525 g, 4 mmol) in water (25 mL), which was pre-cooled to 0–5 °C. After 20 min of stirring the reaction mixture was let to stand at 5 °C for 6 h. Yield 0.81 g (85%) yellow solid m.p. 185–186 °C; ^1^H-NMR (400 MHz, DMSO-*d*_6_): δ 7.36 (td, *J* = 7.5, 1.1 Hz, 1H, Ar), 7.72–7.77 (m, 1H, Ar), 7.84–7.88 (m, 2H,Ar), 11.22–11.93 (bs, 2H); ^13^C-NMR (101 MHz, DMSO-*d*_6_): δ 101.9, 108.0, 108.5, 110.2, 116.9, 119.8, 126.0, 134.1, 134.3, 144.0, 161.7; IR (cm^−1^): 1291, 1432, 1445, 1490, 1607, 1703, 2216 (CN), 2255 (CN), 2298 (CN), 3200; HRMS (HESI *m/z*): [M + H]^+^ calcd for C_11_H_7_N_6_O 239.0676; found 239.0675.

#### 3.2.2. General Procedure for the Preparation of 2-Aryl-3-imino-5-oxo-2,3,4,5-tetrahydro-1,2,4- triazine-6-carbonitriles 4

Corresponding arylhydrazone **3** (2 mmol) was suspended in mixture of ethanol-water (3:1; 25 mL) and resulting mixture was refluxed with stirring for 60 min. After this time, water was added to the hot mixture (15 mL) and allowed to cool down. The precipitated crystalline compound was filtered off, washed with water and dried at 80 °C. For the highest purity it is possible to crystalize the prepared compounds **4** from ethanol.

*3-Imino-5-oxo-2-phenyl-2,3,4,5-tetrahydro-1,2,4-triazine-6-carbonitrile* (**4a**). Yield 88% as beige crystalline solid, m.p. 238–242 °C. ^1^H-NMR (400 MHz, DMSO-*d*_6_): δ 6.47–7.47 (bs, 1H,), 7.55–7.62 (m, 5H, Ar), 7.74–8.62 (bs, 1H); ^13^C-NMR (101 MHz, DMSO-*d_6_*): δ 113.5, 125.7, 126.9, 130.1, 130.4, 139.0, 154.7, 159.2; IR (cm^−1^): 769, 1202, 1415, 1567, 1664, 2231 (CN), 3085; HRMS (HESI *m/z*): [M + H]^+^ calcd for C_10_H_8_N_5_O 214.0723; found 214.0724.

*3-Imino-2-(4-methylphenyl)-5-oxo-2,3,4,5-tetrahydro-1,2,4-triazine-6-carbonitrile* (**4b**). Yield 92% as light yellow crystalline solid, m.p. 246–250 °C. ^1^H-NMR (400 MHz, DMSO-*d*_6_): δ 2.39 (s, 3H, CH_3_), 6.70–7.19 (bs, 1H), 7.38–7.40 (m, 2H, Ar), 7.43–7.45 (m, 2H, Ar), 7.74–8.59 (bs, 1H); ^13^C-NMR (101 MHz, DMSO-*d*_6_): δ 20.9, 113.6, 125.7, 126.7, 130.6, 136.6, 140.1, 154.8, 159.2; IR (cm^−1^): 787, 1428, 1516, 1562,1659, 2236 (CN), 3099; HRMS (HESI *m/z*): [M + H]^+^ calcd for C_11_H_10_N_5_O 228.0880; found 228.0879.

*3-Imino-2-(4-methoxyphenyl)-5-oxo-2,3,4,5-tetrahydro-1,2,4-triazine-6-carbonitrile* (**4c**). Yield 93% as light yellow crystalline solid, m.p. 285–289 °C. ^1^H-NMR (400 MHz, DMSO-*d*_6_): δ 3.82 (s, 3H, CH_3_), 6.58–7.09 (bs, 1H), 7.10–7.16 (m, 2H, Ar), 7.47–7.51 (m, 2H, Ar), 7.75–8.45 (bs, 1H); ^13^C-NMR (101 MHz, DMSO-*d*_6_): δ 55.6, 113.6, 115.2, 125.6, 128.4, 131.7, 155.1, 159.2, 160.3; IR (cm^−1^): 999, 1175, 1244, 1511, 1636, 2236 (CN), 3131; HRMS (HESI *m/z*): [M + H]^+^ calcd for C_11_H_10_N_5_O_2_ 244.0829; found 244.028.

*2-(4-Chlorophenyl)-3-imino-5-oxo-2,3,4,5-tetrahydro-1,2,4-triazine-6-carbonitrile* (**4d**). Yield 89% as light yellow crystalline solid, m.p. 249–253 °C. ^1^H-NMR (400 MHz, DMSO-*d*_6_): δ 6.87–7.52 (bs, 1H), 7.60–7.63 (m, 2H, Ar), 7.65–7.68 (m, 2H, Ar) 7.68–7.79 (bs, 1H); ^13^C-NMR (101 MHz, DMSO-*d_6_*): δ 113.4, 125.8, 128.9, 130.2, 135.0, 137.8, 154.7, 159.1; IR (cm^−1^): 856, 1018, 1294, 1405, 1511, 1637, 2236 (CN), 3140; HRMS (HESI *m/z*): [M + H]^+^ calcd for C_10_H_7_ClN_5_O 248.0334; found 248.0335.

*3-Imino-2-(4-nitrophenyl)-5-oxo-2,3,4,5-tetrahydro-1,2,4-triazine-6-carbonitrile* (**4f**). Yield 89% as light yellow crystalline solid, m.p. 238–242 °C. ^1^H-NMR (400 MHz, DMSO-*d*_6_): δ 6.85-7.75 (bs, 1H), 7.87–7.91 (m, 2H, Ar), 8.43–8.46 (m, 2H, Ar) ^13^C-NMR (101 MHz, DMSO-*d*_6_): δ 113.4, 125.6, 126.2, 128.7, 144.0, 148.2, 154.4, 159.0; IR (cm^−1^): 856, 1189, 1350, 1535, 1682, 2227 (CN), 3171; HRMS (HESI *m/z*): [M + H]^+^ calcd for C_10_H_7_N_6_O_3_ 259.0574; found 259.0572.

*3,5-Dioxo-2-phenyl-2,3,4,5-tetrahydro-1,2,4-triazine-6-carbonitrile* (**5**). Nitrile **4a** (200 mg; 0.94 mmol) was refluxed in a mixture of ethanol (10 mL), water (10 mL) and aqueous 10% hydrochloric acid (3 mL) for 24 h. After this time the reaction mixture was concentrated under reduced pressure to one half of its original volume, when the product started to crystalize. The product was filtered off, washed with water and dried. Yield 134 mg (67%) as a colorless crystalline solid, m.p. 244–246 °C (243–245 °C [12,15]). ^1^H-NMR (400 MHz, DMSO-*d_6_*): δ 7.45–7.55 (m, 5H, Ar), 13.03 (s, 1H); ^13^C-NMR (101 MHz, DMSO-*d*_6_): δ 112.6, 122.4, 125.8, 129.00, 129.02, 139.3, 147.2, 154.8; IR (cm^−1^): 575, 769, 1139, 1535, 1714, 2240 (CN), 2826, 3057; HRMS (HESI *m/z*): [M − H]^−^ calcd for C_10_H_5_N_4_O_2_ 213.0418; found 213.0405.

*3,5-Dioxo-2-phenyl-2,3,4,5-tetrahydro-1,2,4-triazine-6-carboxylic acid* (**6**). Nitrile **4a** (100 mg; 0.47 mmol) was refluxed in aqueous 20% hydrochloric acid (4 mL) for 2 h. After this time, the reaction mixture was concentrated under reduced pressure to one third of its original volume, when the product started to crystalize. The product was filtered off, washed with water and dried. Yield 96 mg (88%) as a colorless crystalline solid, m.p. 222–226 °C (224–226 °C [12,21]). ^1^H-NMR (400 MHz, DMSO-*d*_6_): δ 7.49–7.53 (m, 5H, Ar), 12.46 (s, 1H), 13.23–13.86 (bs, 1H); ^13^C-NMR (101 MHz, DMSO-*d*_6_): δ 125.9, 128.4, 128.7, 135.8, 139.7, 147.8, 154.6, 161.8. HRMS (HESI *m/z*): [M − H]^−^ calcd for C_10_H_6_N_3_O_4_ 232.0364; found 232.0354.

*4-Oxo-1,6-diphenyl-1,4-dihydroimidazo[2,1-c][1,2,4]triazine-3-carbonitrile* (**8**). A mixture of nitrile **4a** (48 mg, 0.23 mmol) and phenacyl bromide (46 mg, 0.23 mmol) in DMF (1.5 mL) was heated at 130 °C for 24 h. Cooled mixture was poured into water (20 mL) and precipitated solid was filtered off, washed with water and dried. Yield 46 mg (65%). The crude product was crystalized from DMF (0.4 mL) to get an orange crystalline compound (30 mg). m.p 244–246 °C. ^1^H-NMR (400 MHz, DMSO-*d*_6_): δ 7.37–7.40 (m, 1H, Ar), 7.44–7.47 (m, 2H, Ar), 7.60–7.64 (m, 1H, Ar), 7.67–7.71 (m, 2H, Ar), 7.92–7.94 (m, 2H, Ar), 7.98–8.01 (m, 2H, Ar), 8.69 (s, 1H, Ar); ^13^C-NMR (101 MHz, DMSO-*d*_6_): δ 106.8, 113.5, 115.0, 124.6, 126.0, 128.9, 129.0, 129.4, 129.8, 131.4, 139.9, 142.2, 143.0, 149.2. HRMS (HESI *m/z*): [M + H]^+^ calcd for C_18_H_12_N_5_O 314.1036; found 314.1035.

*3,6-Dioxo-4,6-dihydro-3H-[1,2,4]triazino[2,3-a]quinazoline-2-carbonitrile* (**9**). Hydrazone **3e** (400 mg, 1.56 mmol) was suspended in mixture of ethanol-water (3:1; 25 mL) and resulting mixture was refluxed with stirring for 24 h. After this time, water was added to the hot mixture (15 mL) and allowed to cool down. The precipitated crystalline compound was filtered off, washed with water and dried at 80 °C. Yield 250 mg (67%), m.p. over 360 °C (367–370 °C [22]). ^1^H-NMR (400 MHz, DMSO-*d*_6_): 7.49–7.53 (m, 1H, Ar), 7.78 (ddd, *J* = 8.7, 7.2, 1.6 Hz, 1H, Ar), 7.98 (d, *J* = 8.0 Hz, 1H, Ar), 8.03 (dd, *J* = 7.8, 1.5 Hz, 1H, Ar); ^13^C-NMR (101 MHz, DMSO-*d*_6_): δ 114.1; 114.8; 119.5; 126.7; 126.8; 127.1; 133.4; 139.1; 151.7; 159.6; 166.3. IR (cm^−1^): 759, 122, 152, 1571, 1638, 2245 (CN); HRMS (HESI *m/z*): [M + H]^+^ calcd for C_11_H_6_N_5_O_2_ 240.0516; found C_11_H_6_N_5_O_2_.

*6-Imino-3-oxo-4,6-dihydro-3H-[1,2,4]triazino[2,3-a]quinazoline-2-carbonitrile* (**10**). Cyanohydrazone **3g** (255 mg, 1.07 mmol) was suspended in a mixture of ethanol-water (3:1; 20 mL) and the resulting mixture was refluxed with stirring for 60 min. After cooling, the precipitated crystalline compound was filtered off, washed with water and dried at 80 °C. Yield 222 mg (87%) beige crystalline solid, m.p. 350–360 °C (decomp). ^1^H-NMR (400 MHz, DMSO-*d*_6_): δ 7.70 (ddd, *J* = 8.2, 7.4, 1.0 Hz, 1H, Ar), 7.99 (ddd, *J* = 8.6, 7.3, 1.3 Hz, 1H, Ar), 8.21 (dd, *J* = 8.6, 0.8 Hz, 1H, Ar), 8.33 (dd, *J* = 8.2, 1.0 Hz, 1H, Ar) 9.13 (s, 1H), 9.23 (s, 1H); ^13^C-NMR (101 MHz, DMSO-*d*_6_): δ 112.2, 113.5, 115.8, 125.0, 127.5, 128.8, 135.6, 138.7, 150.8, 160.0, 161.2; IR (cm^−1^): 759, 1175, 1354, 1444, 1492, 1567, 2241 (CN), 3087; HRMS (HESI *m/z*): [M + H]^+^ calcd C_11_H_7_N_6_O 239.0676; found 239.0676.

*6-Imino-3-oxo-4,6-dihydro-3H-[1,2,4]triazino[2,3-a]quinazoline-2-carboxylic acid* (**11**). Nitrile **10** (50 mg, 0.21 mmol) was suspended in a mixture of aqueous hydrochloric acid (1:1, 4 mL) and refluxed for 20 min. Formed solution was cooled and neutralized with diluted ammonium hydroxide (1:2) to pH = 3–4. Precipitated white microcrystalline solid was filtered off, washed with water and dried. Yield 44 mg (82%), m.p. 252–254 °C (decomp). ^1^H-NMR (400 MHz, DMSO-*d*_6_): δ 7.65–7.69 (m, 1H, Ar), 7.96–8.00 (m, 1H, Ar), 8.24 (d, *J* = 8.4 Hz, 1H, Ar), 8.31 (d, *J* = 7.9 Hz, 1H, Ar), 9.12 (s, 1H), 9.21 (s, 1H); 15.09–15.38 (bs, 1H, COOH); ^13^C-NMR (101 MHz, DMSO-*d*_6_): δ 112.4, 116.0, 125.0, 127.4, 135.6, 139.0, 139.2, 151.5, 161.4, 162.4, 163.7. HRMS (HESI *m/z*): [M − H]^−^ calcd for C_11_H_6_N_5_O_3_ 256.0476; found 256.0475.

*3,6-Dioxo-4,6-dihydro-3H-[1,2,4]triazino[2,3-a]quinazoline-2-carboxylic acid* (**12**). Nitrile **10** (166 mg, 0.7 mmol) was suspended in concentrated hydrochloric acid (4.5 mL) and refluxed for 24 h. After this time, the precipitated white crystalline solid was filtered off, washed with water and dried. Yield 126 mg (70%), m.p. 256–258 °C (decomp) (255–257 °C [22]). ^1^H-NMR (400 MHz, DMSO-*d*_6_): δ 7.60–7.64 (m, 1H, Ar), 7.96 (ddd, *J* = 8.6, 7.3, 1.5 Hz, 1H, Ar), 8.07 (d, *J* = 8.3 Hz, 1H, Ar), 8.14 (dd, *J* = 7.9, 1.3 Hz, 1H, Ar), 12.66–13.28 (bs, 1H), 13.81–14.90 (bs, 1H); ^13^C-NMR (101 MHz, DMSO-*d*_6_): δ 115.4, 117.7, 127.3, 127.4, 135.9, 138.8, 143.2, 149.0, 159.1, 159.4, 162.8. HRMS (HESI *m/z*): [M − H]^−^ calcd for C_11_H_5_N_4_O_4_ 257.0305; found 257.0309.

*3-Cyano-1,5-bis(2-cyanophenyl)formazan* (**13g**). a) This compound was prepared according to Method A with different diazotation process. A solution of 2-aminobenzencabonitrile (0.472 g, 4 mmol) in a mixture of acetic acid (10.5 mL) and 35% hydrochloric acid (3 mL) was diazotized at 0–5 °C with a solution of sodium nitrite (0.275 g, 4 mmol) in ice water (3 mL). The mixture was stirred in ice bath for 1 h and then added portionwise to a solution of 2-cyanoacetylcyanamide sodium salt **2** (0.525 g, 4 mmol), sodium acetate (3 g) in water (25 mL), which was pre-cooled to 0–5 °C. After 5 h of stirring the precipitated red solid was filtered off, washed with water and dried. A sample for analysis was crystallized from ethanol (0.1 g/10 mL). Yield 510 mg (85%, based on 2-aminobenzencabonitrile), m.p. 187–191 °C (190 °C [19]) as red crystals. ^1^H-NMR (400 MHz, CDCl_3_): δ 7.44–7.48 (td, *J* = 7.6, 1.0 Hz, 2H, Ar), 7.71–7.77 (m, 2H, Ar), 7.94 (d, *J* = 8.3 Hz, 2H, Ar), 12.33 (s, 1H); ^13^C-NMR (101 MHz, CDCl_3_): δ 104.5, 112.0, 116.6, 116.8, 118.3, 120.8, 125.2, 127.6, 129.4, 132.5, 133.1, 133.5, 134.2, 134.3, 134.4, 134.9, 147.9. HRMS (HESI *m*/*z*): [M − H]^−^ calcd for C_16_H_8_N_7_ 298.0836; found 298.0838.

b) Product with the same spectral and physical properties was obtained using cyanoacetic acid in a similar manner as described above. Cyanoacetic acid (42.5 mg, 0.5 mmol), 2-aminobenzencabonitrile (118 mg, 1 mmol), were used for the reaction. Yield 80%.

## 4. Conclusions

In this work, we extended and modified a previously developed method for the preparation of novel 2-aryl-3-imino-5-oxo-2,3,4,5-tetrahydro-1,2,4-triazine-6-carbonitriles (**4-A**), which at high temperature occur as their 3-amino tautomers (**4-B**). These compounds are forms of 1-aryl-6-azaisocytosine derivatives, which to date have not been well studied. The method was based on the coupling of diazonium salts with cyanoacetylcyanamide **2** carried out in slightly basic or acidic conditions to obtain the corresponding 2-arylhydrazono-2-cyanoacetylcyanamides **3**, which were cyclized by heating in polar solvents containing water to form the 1-aryl-6-azaisocytosines **4**. The amino (imino) group at position 3 of the 1,2,4-triazine ring of the model phenyl derivative **4a** was used for the preparation of the novel imidazo[2,1-*c*][1,2,4]triazine **8** and 1,2,4-triazino[2,3-*a*]quinazoline derivatives (**9**–**12**), potentially interesting as model compounds for intercalation testing with DNA.

## Figures and Tables

**Table 1:** Comparison of Methods A and B for the reaction of diazonium salts with compound **2**.

Diazonium Salt Ar-N_2_^+^	Arylhydrazone 3 [Isolated Yield %]	
	Method A	Method B
Ar: phenyl	**3a** [80%]	**3a** [5%] *
Ar: 4-methylphenyl	**3b** [87%]	**3b** [0%]
Ar: 4-methoxyphenyl	**3c** [83%]	**3c** [0%]
Ar: 4-chlorophenyl	**3d** [79%]	**3d** [5%] *
Ar: 2-carboxyphenyl	**3e** [72%]	**3e** [50%]
Ar: 4-nitrophenyl	**3f** [15%] *	**3f** [93%]
Ar: 2-cyanophenyl	**3g** [10%] *	**3g** [80%]

* yield based on HPLC analysis.

## Supplementary Materials

The copies of ^1^H- and ^13^C-NMR spectra are available online.
