# Supplementary material for: General Synthesis of 1-Aryl-6-azaisocytosines and Their Utilization for the Preparation of Related Condensed 1,2,4-Triazines
Source: Molecules. 2019 Oct 1;24(19):3558. doi: 10.3390/molecules24193558 (PMC6804241; doi:10.3390/molecules24193558)

Supporting Information

General Synthesis of 1-Aryl-6-azaisocytosines and their Utilization for the Preparation of Related Condensed 1,2,4-triazines

František Zálešák [1], Jan Slouka [1], Jakub Stýskala [1, *]

[1]Department of Organic Chemistry, Palacky University, 17. listopadu 12, 771 46 Olomouc, Czech Republic

*E-mail: jakub.styskala@upol.cz

^{1}H spectrum of 2:

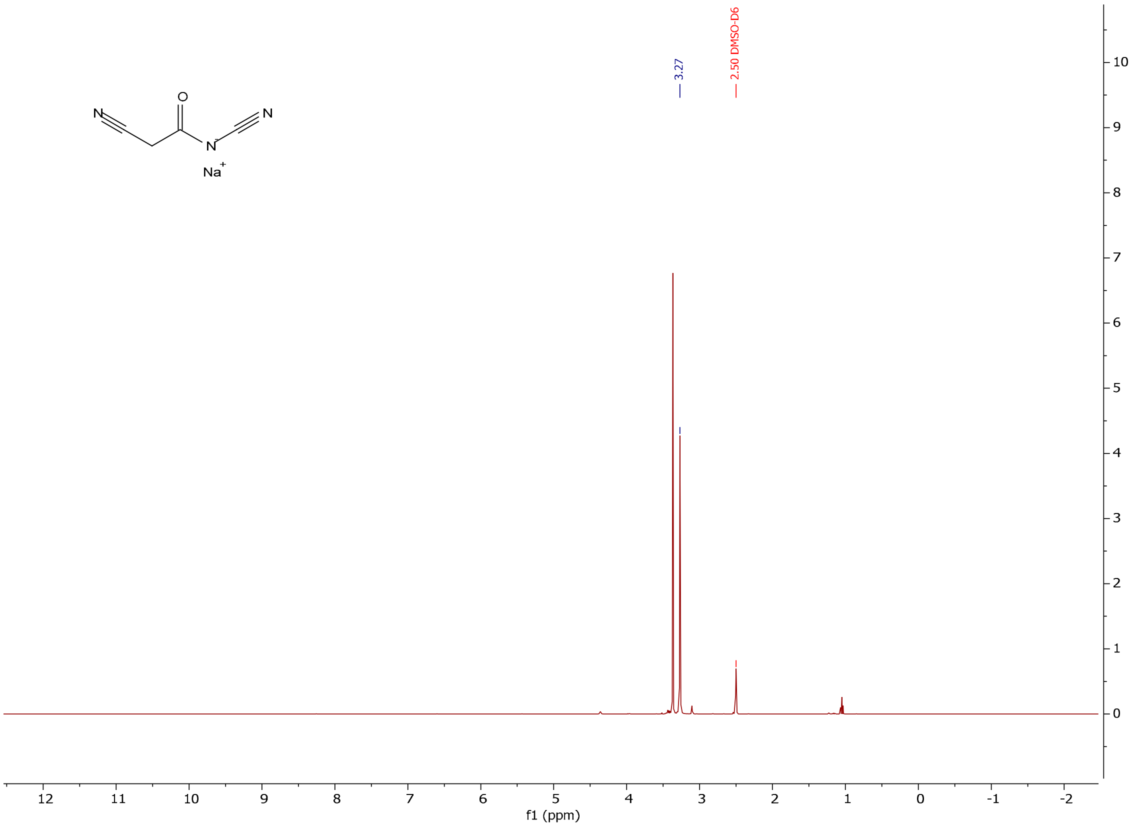

^{13}C spectrum of 2:

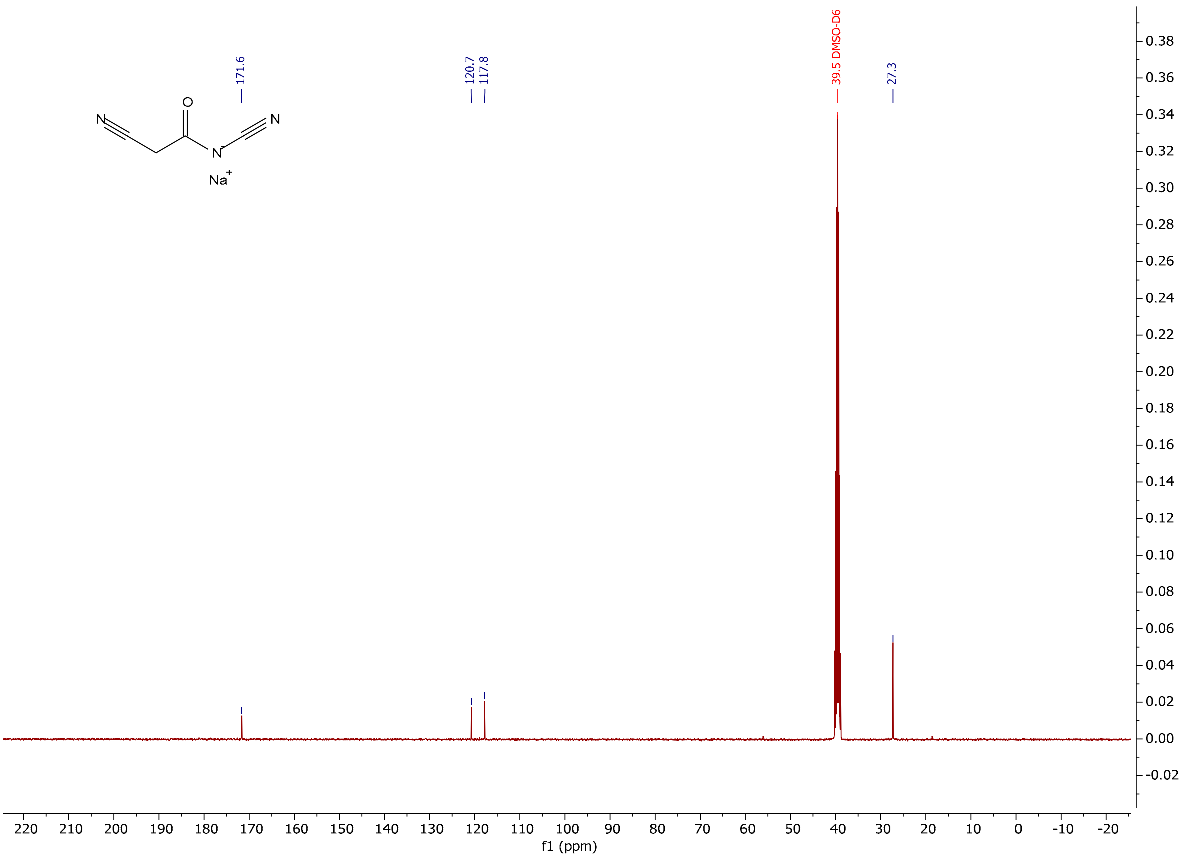

^{1}H spectrum of 3a:

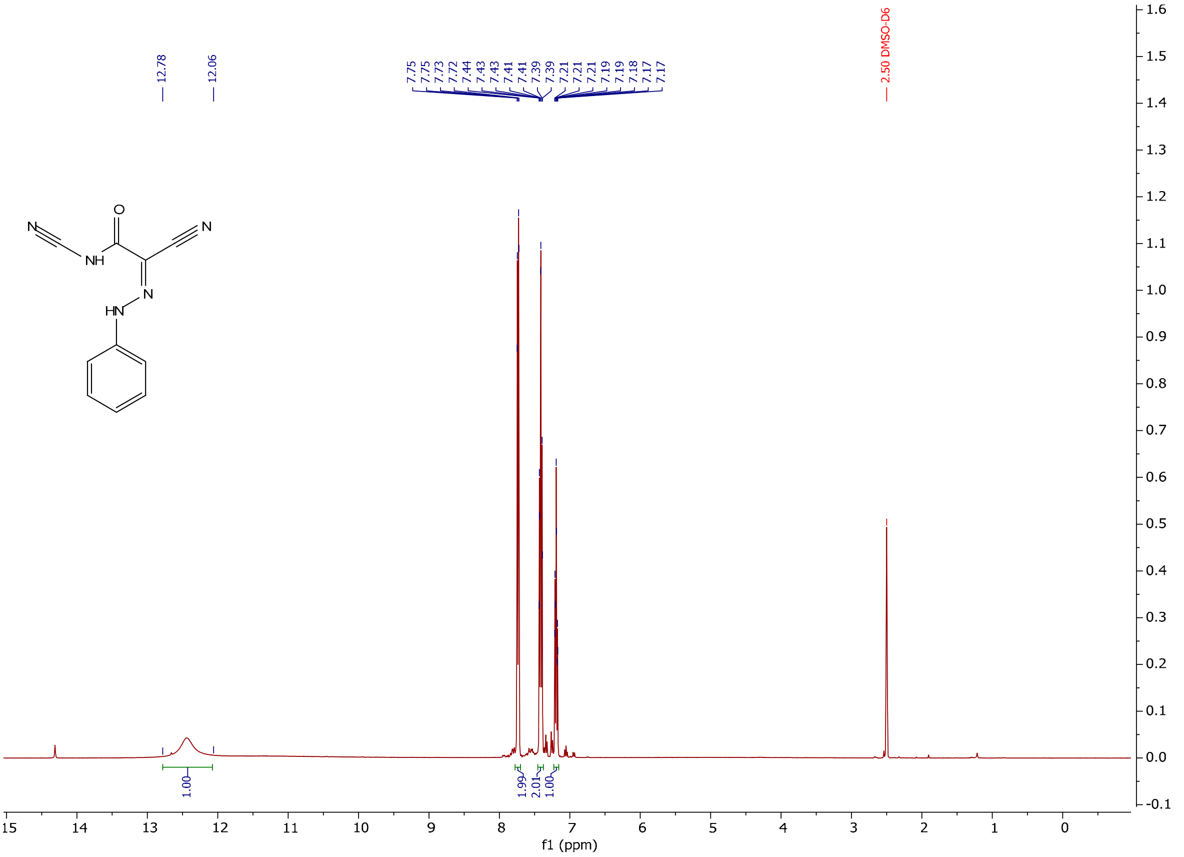

^{13}C spectrum of 3a:

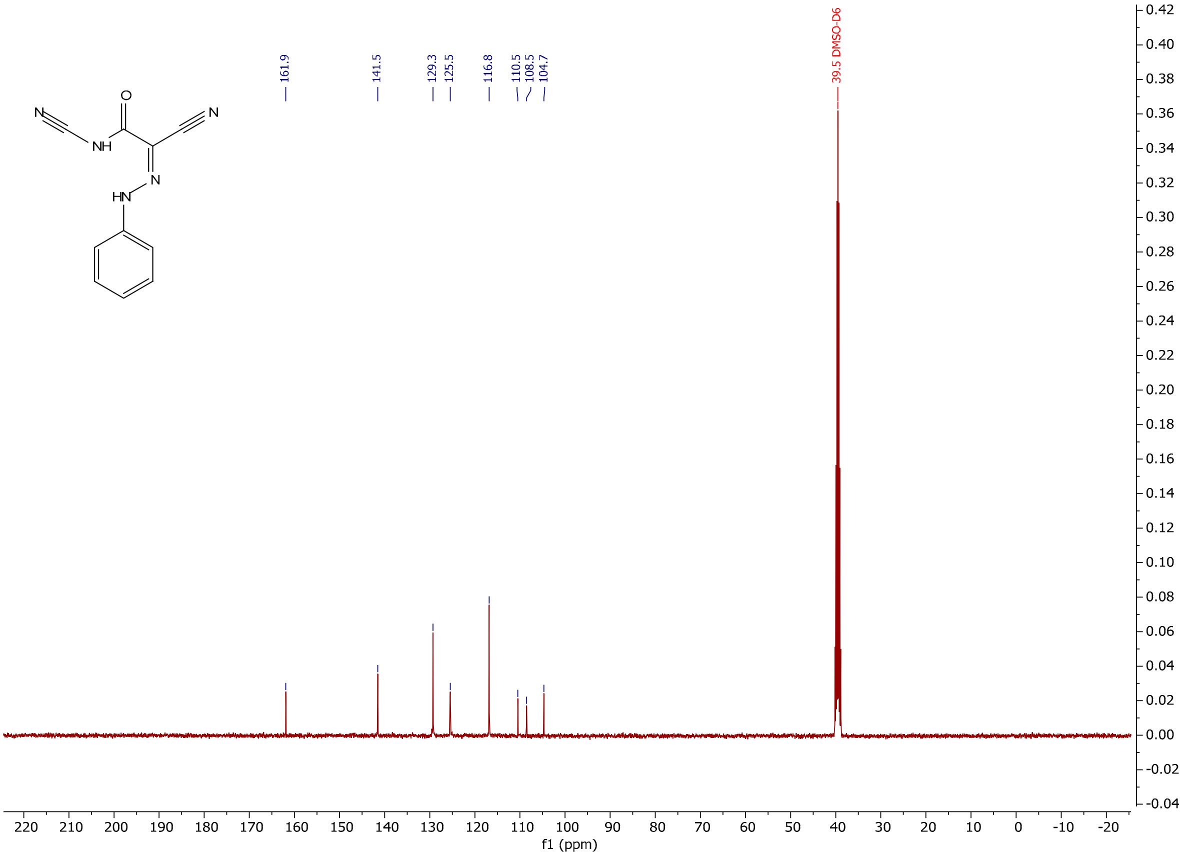

^{1}H spectrum of 3b:

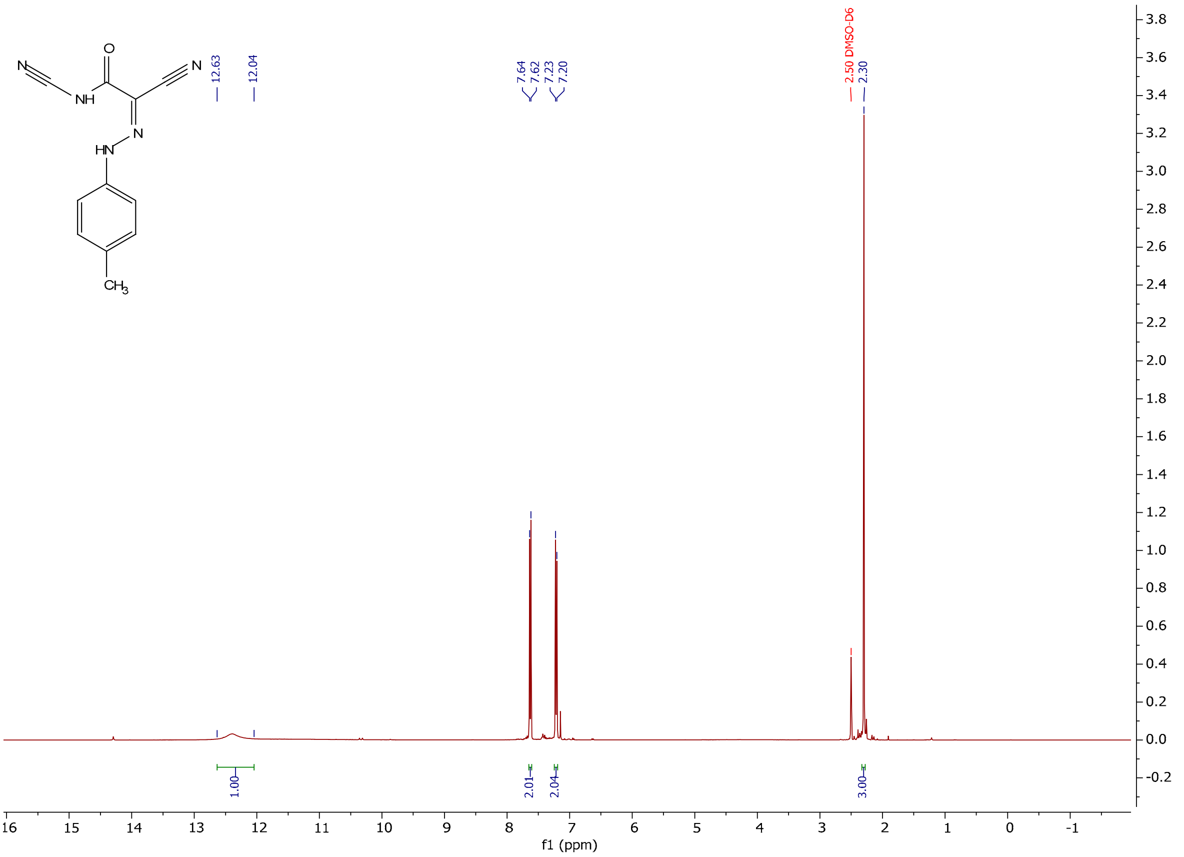

^{13}C spectrum of 3b:

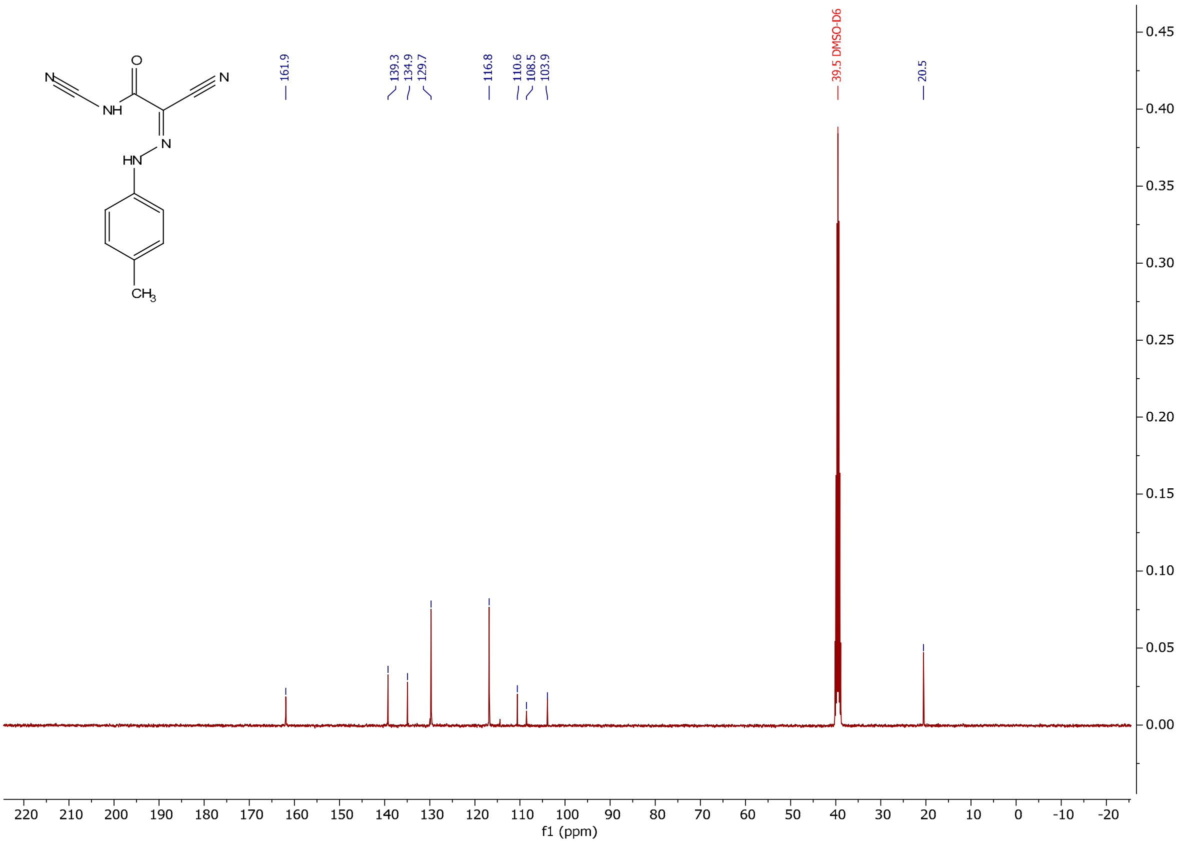

^1H spectrum of 3c:

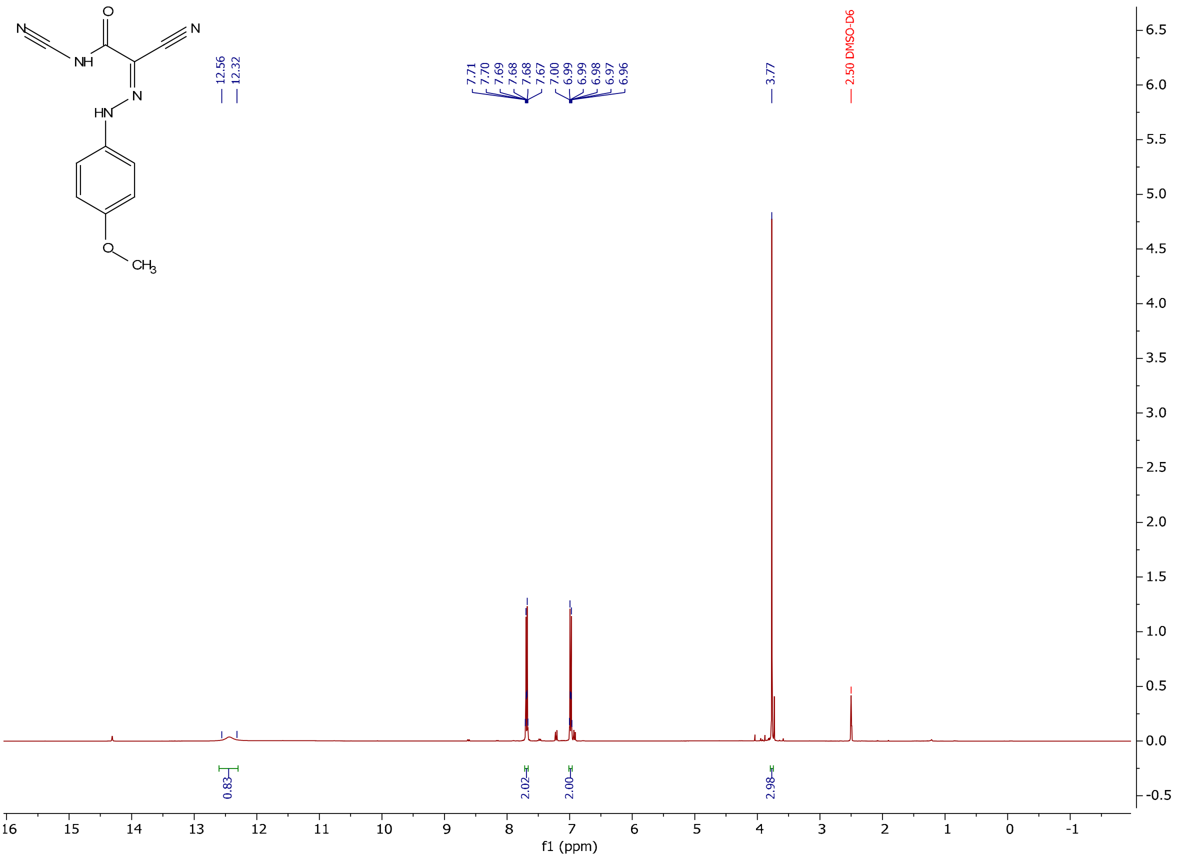

^{13}C spectrum of 3c:

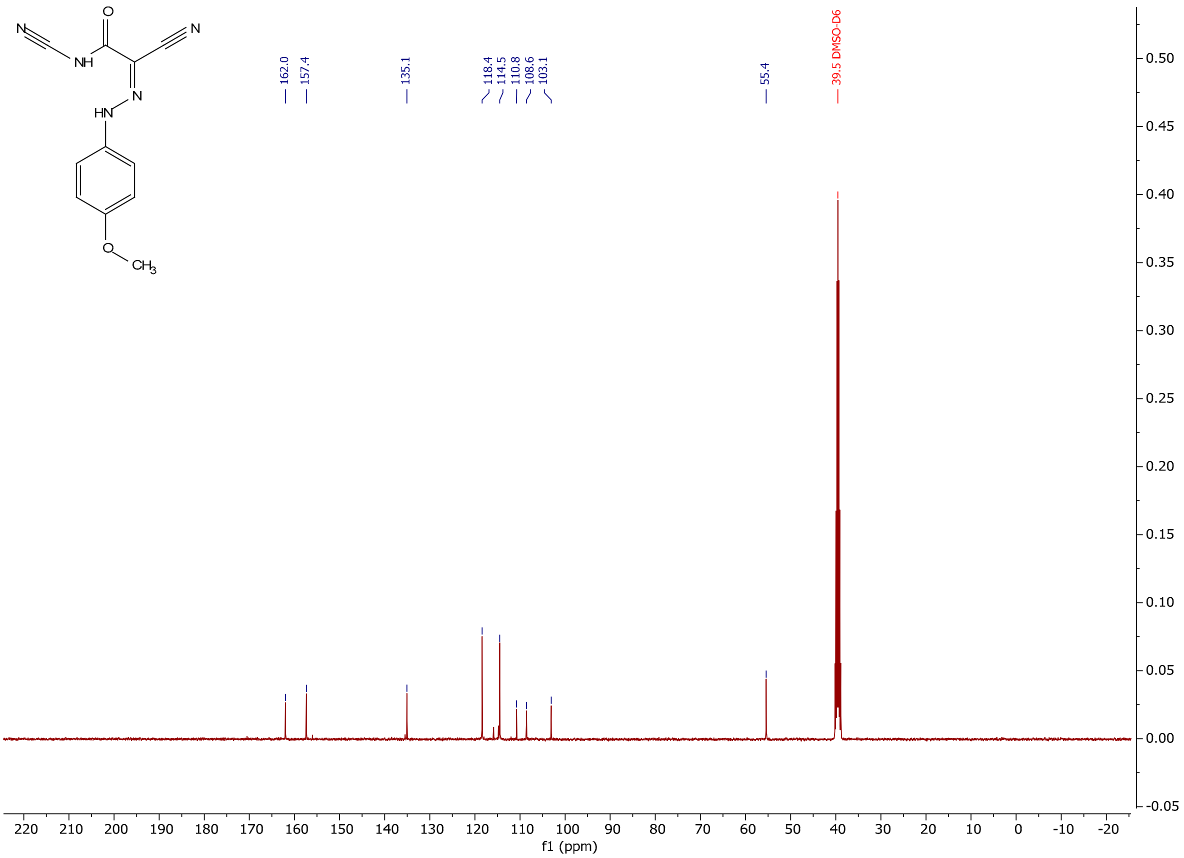

^1H spectrum of 3d:

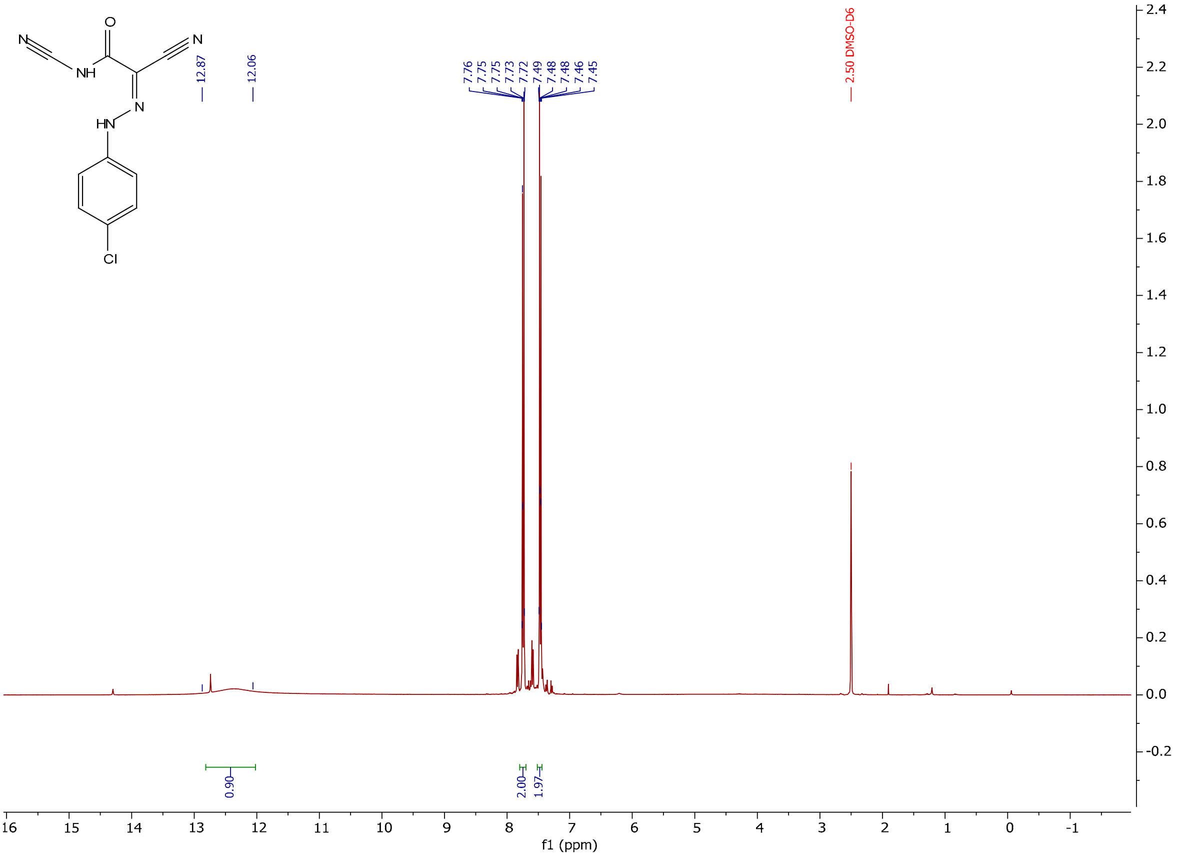

^{13}C spectrum of 3d:

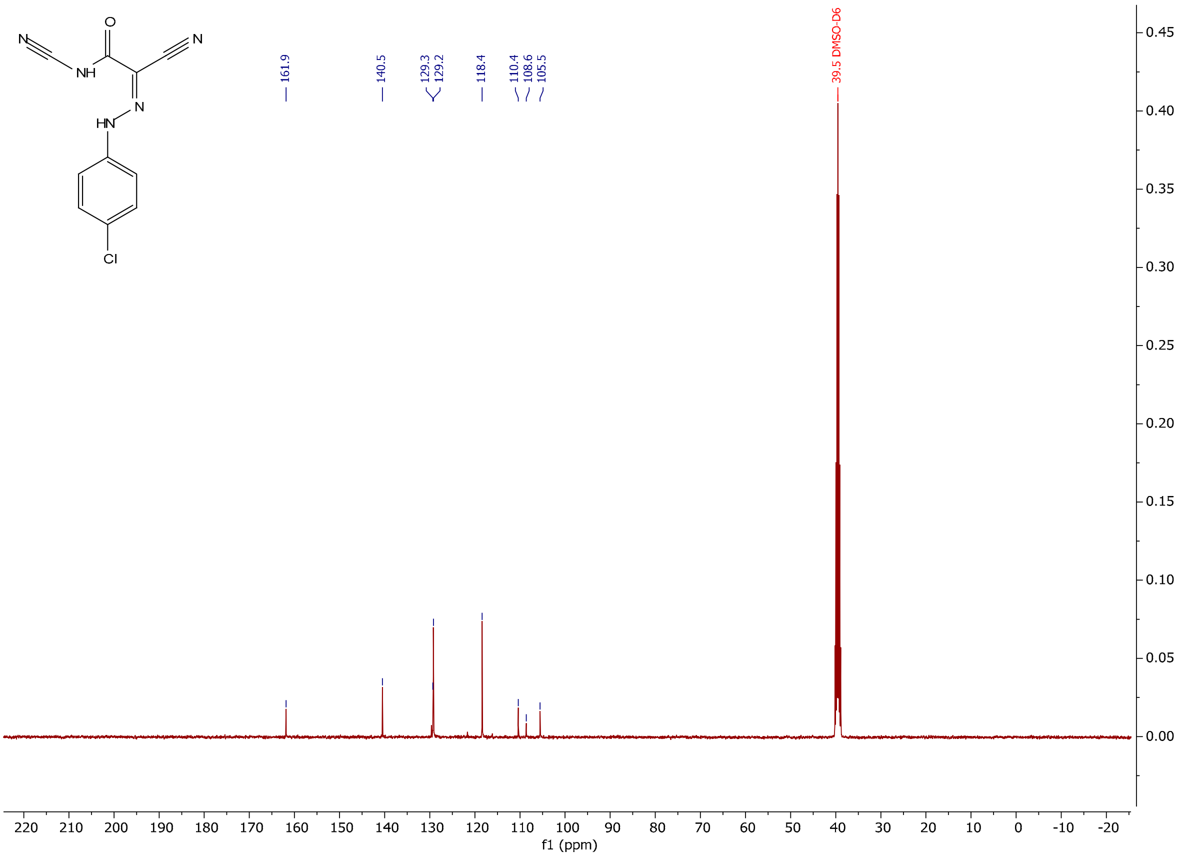

^{1}H spectrum of 3e:

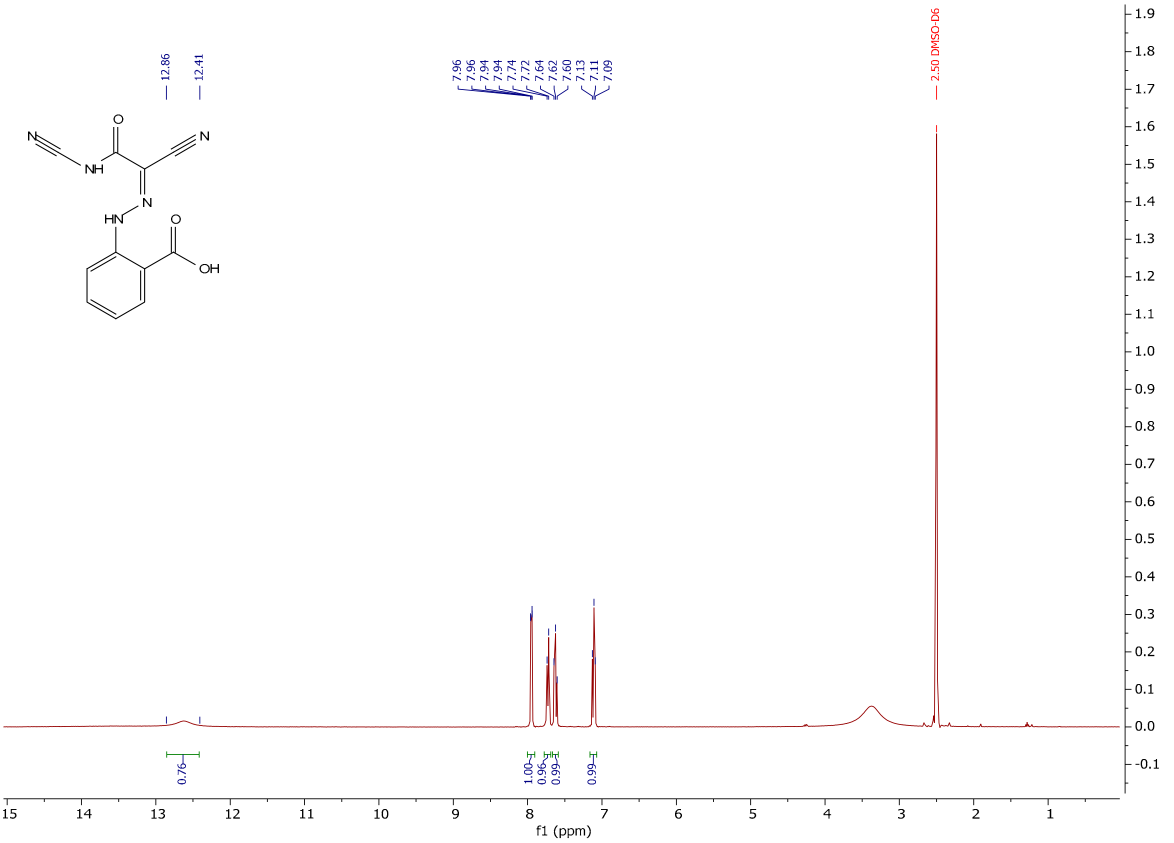

^{13}C spectrum of 3e:

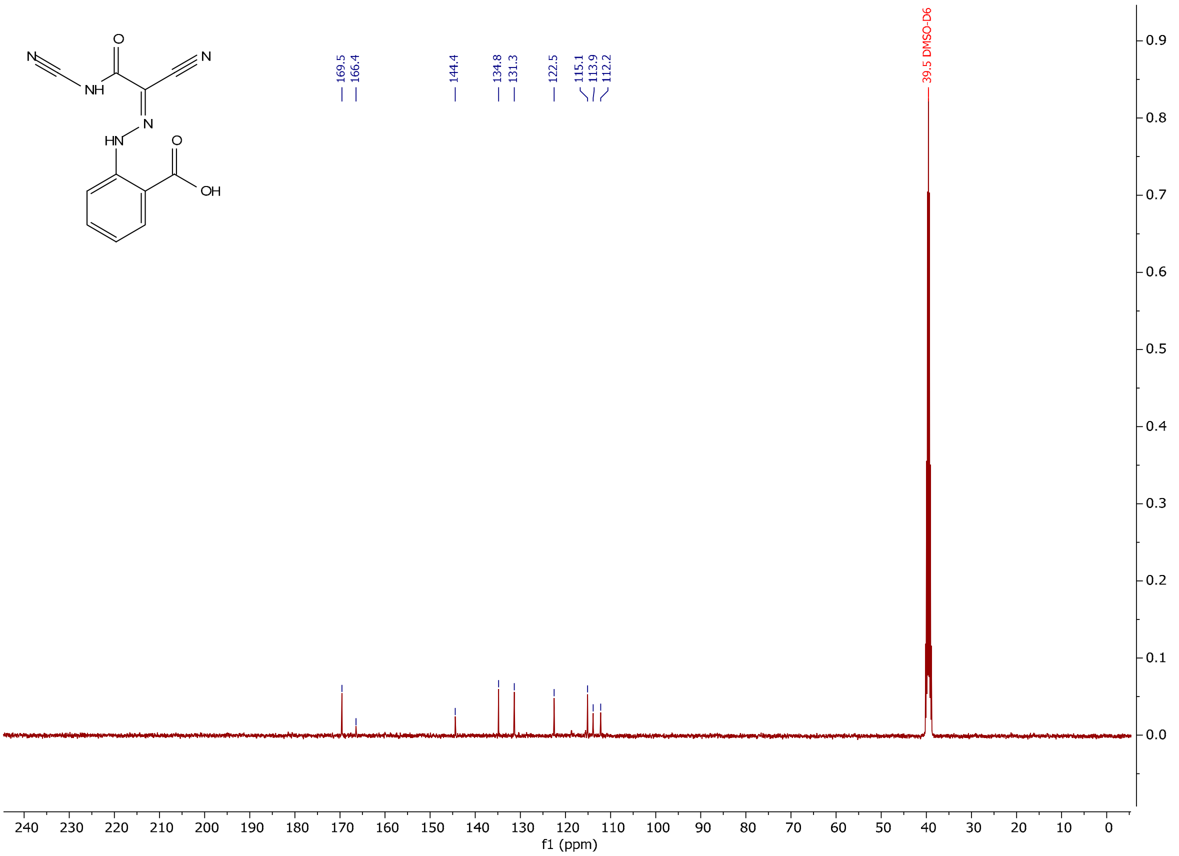

^{1}H spectrum of 3f:

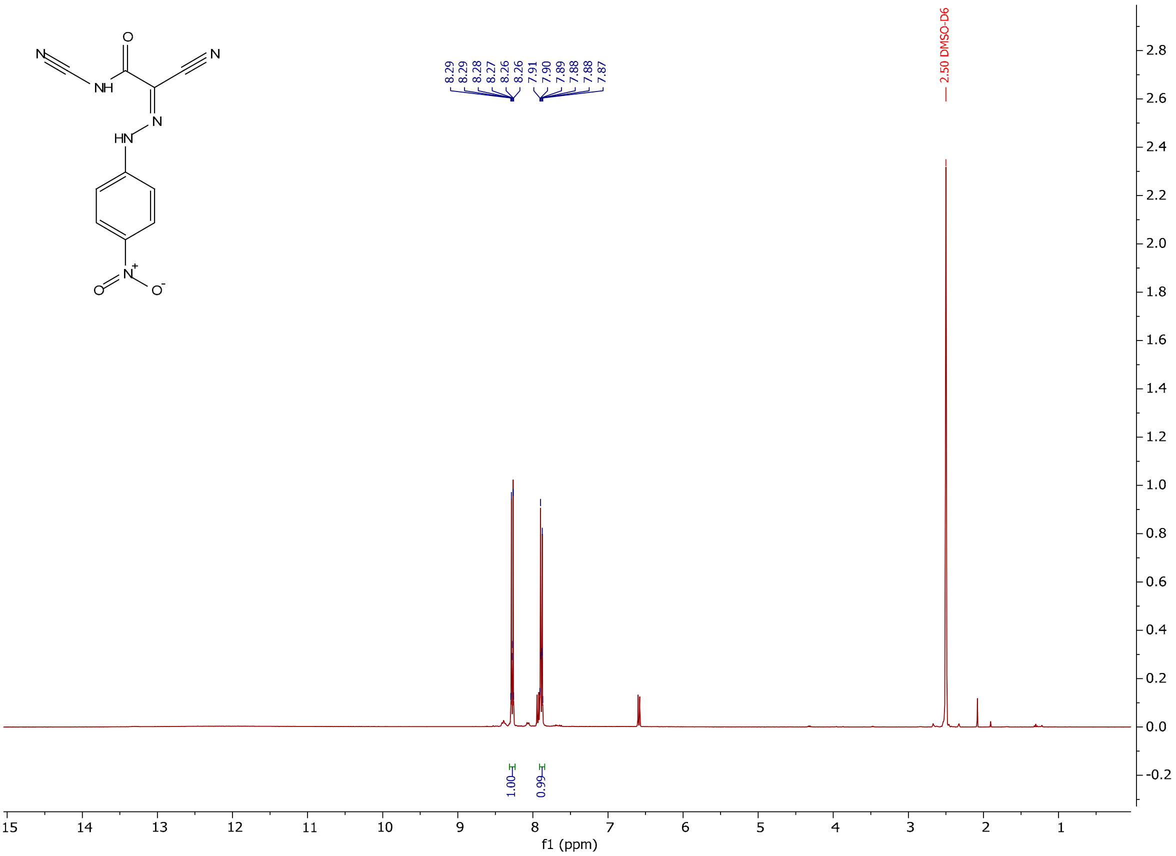

^{13}C spectrum of 3f:

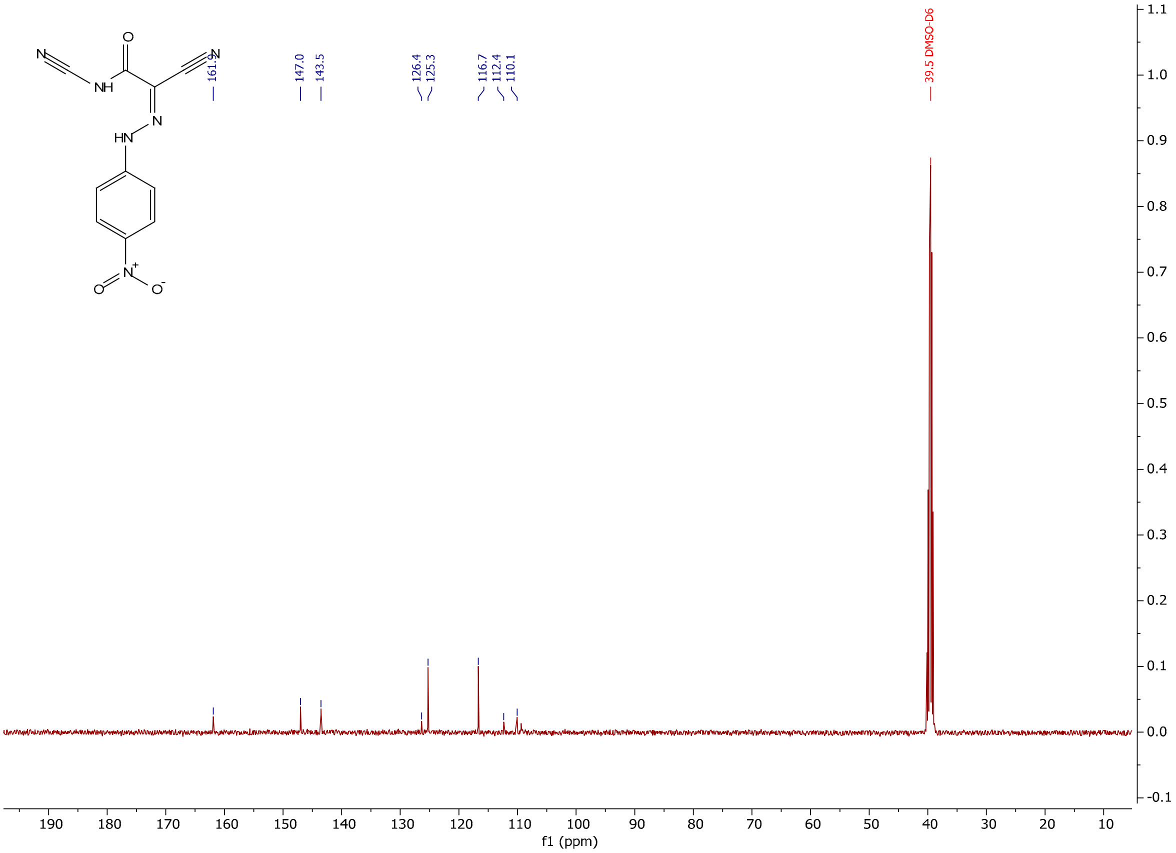

^{1}H spectrum of 3g:

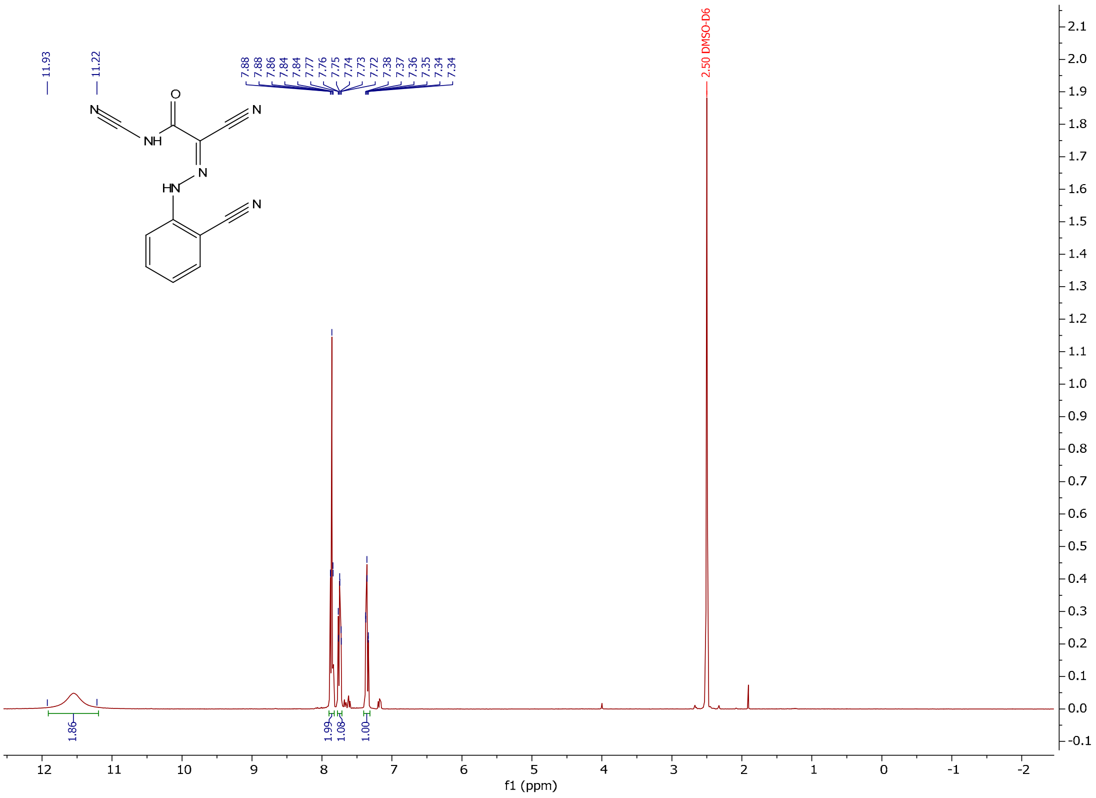

^{13}C spectrum of 3g:

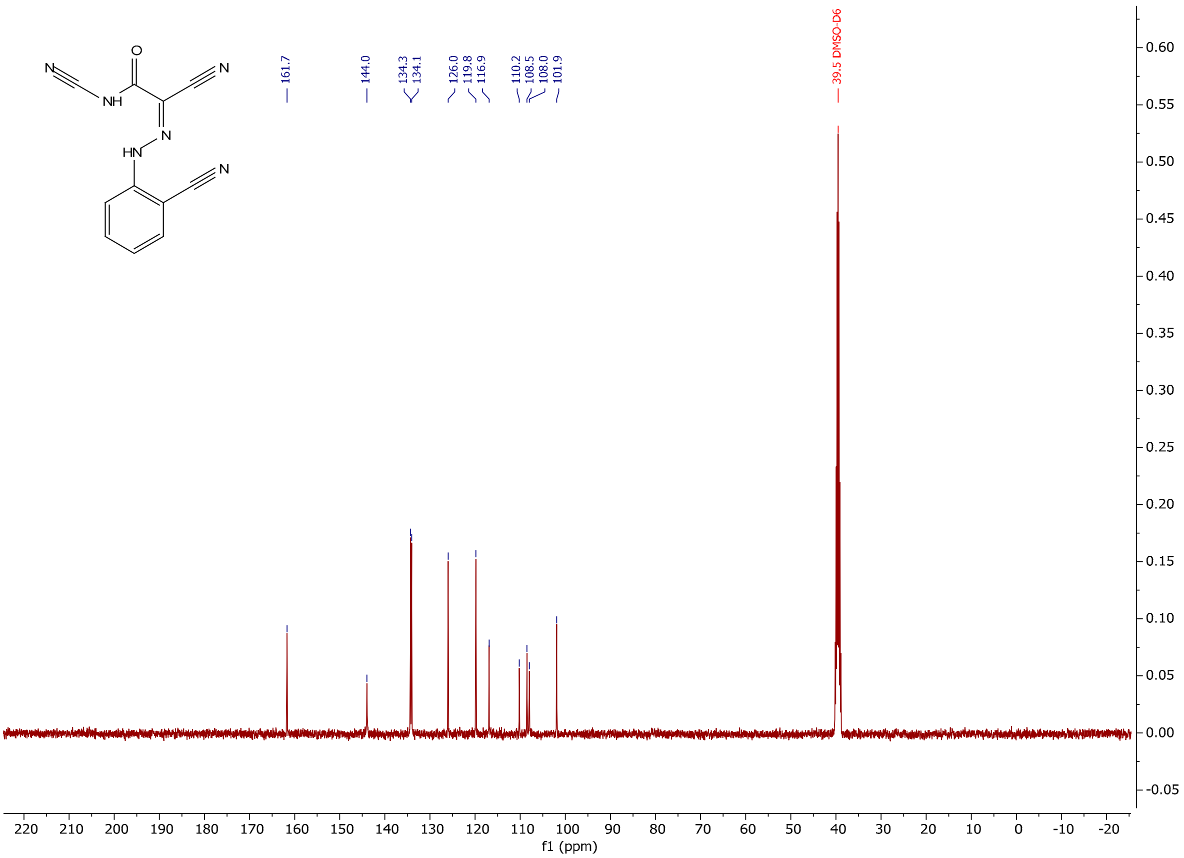

^{1}H spectrum of 4a (recorded at room temperature):

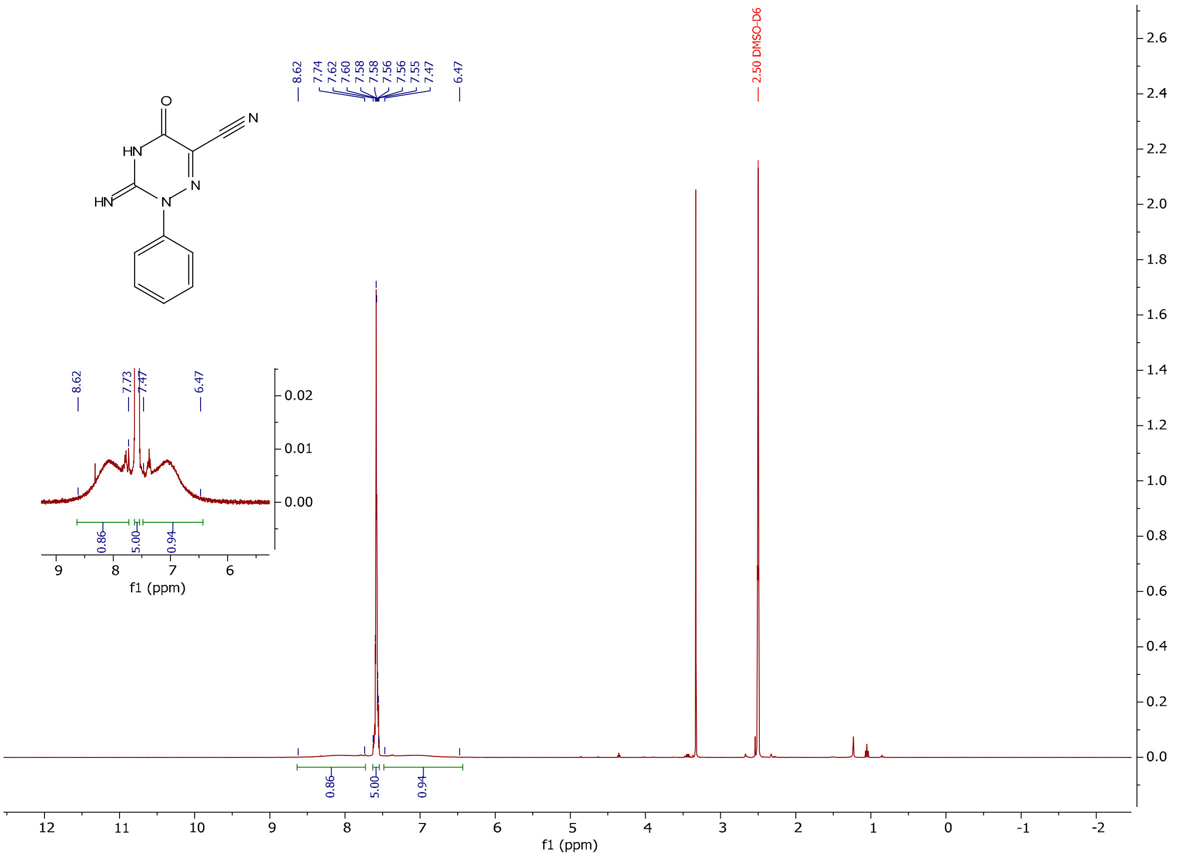

^{13}C spectrum of 4a (recorded at room temperature):

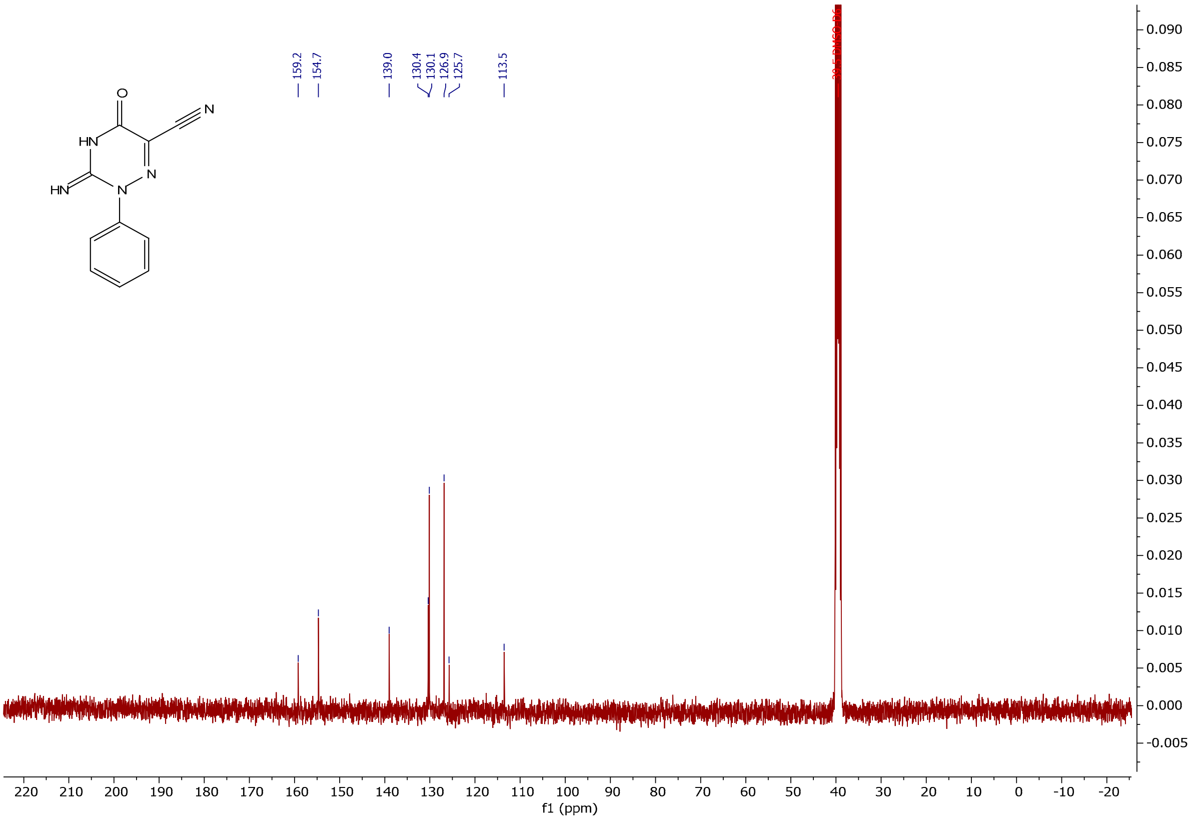

^{1}H spectrum of 4a (recorded at 80 °C):

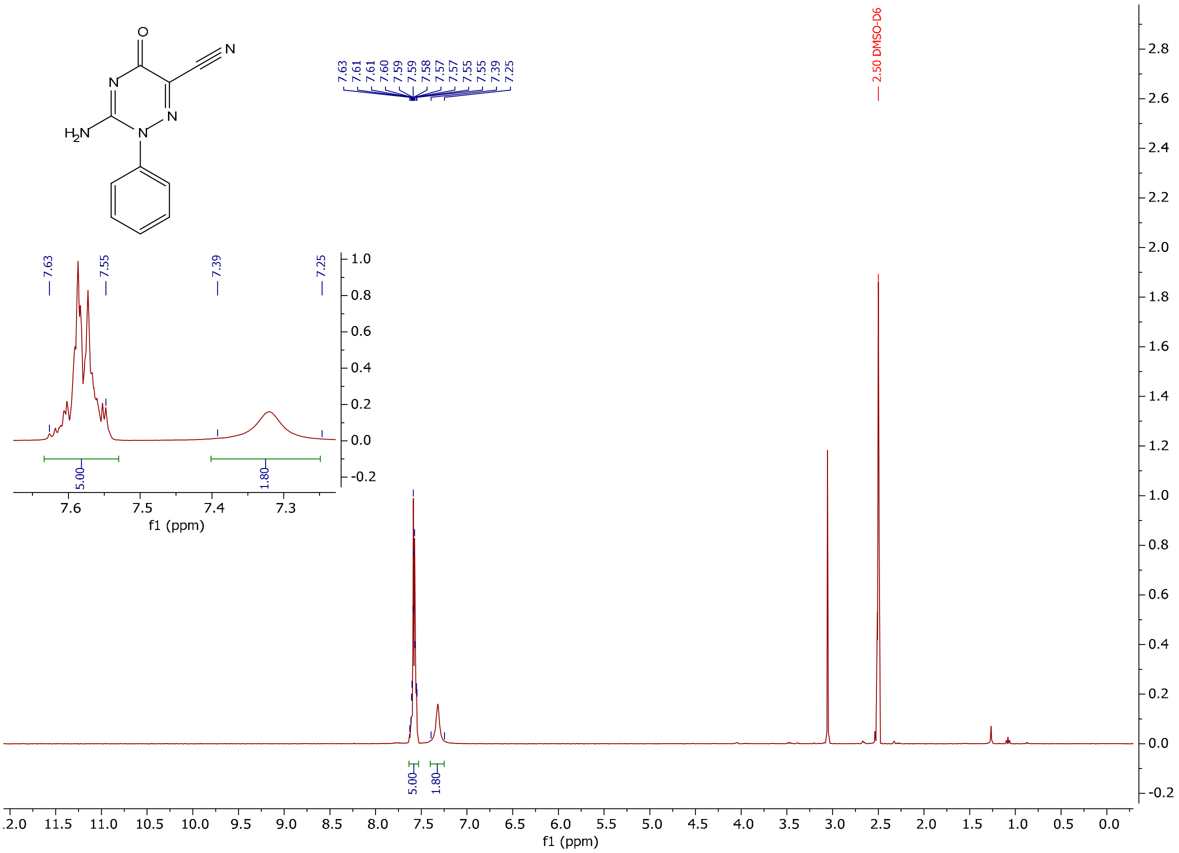

^{1}H spectrum of 4b:

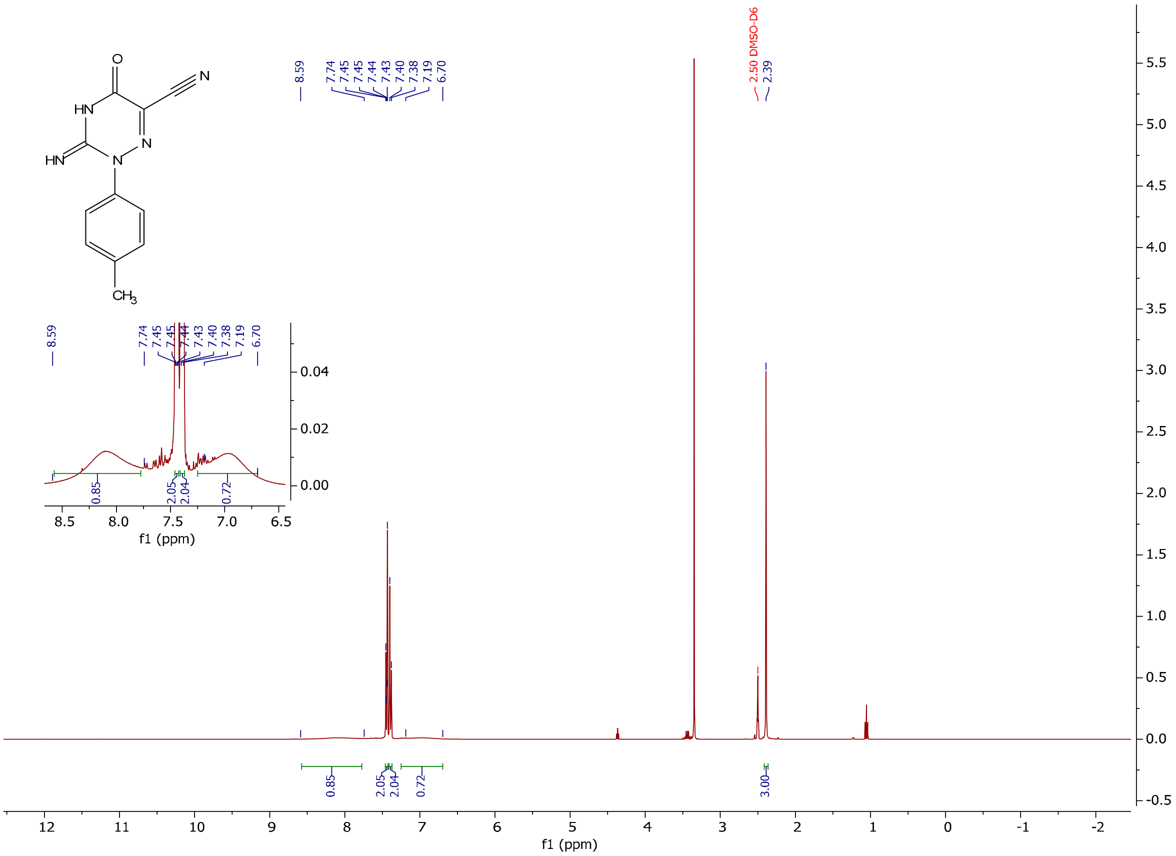

^{13}C spectrum of 4b:

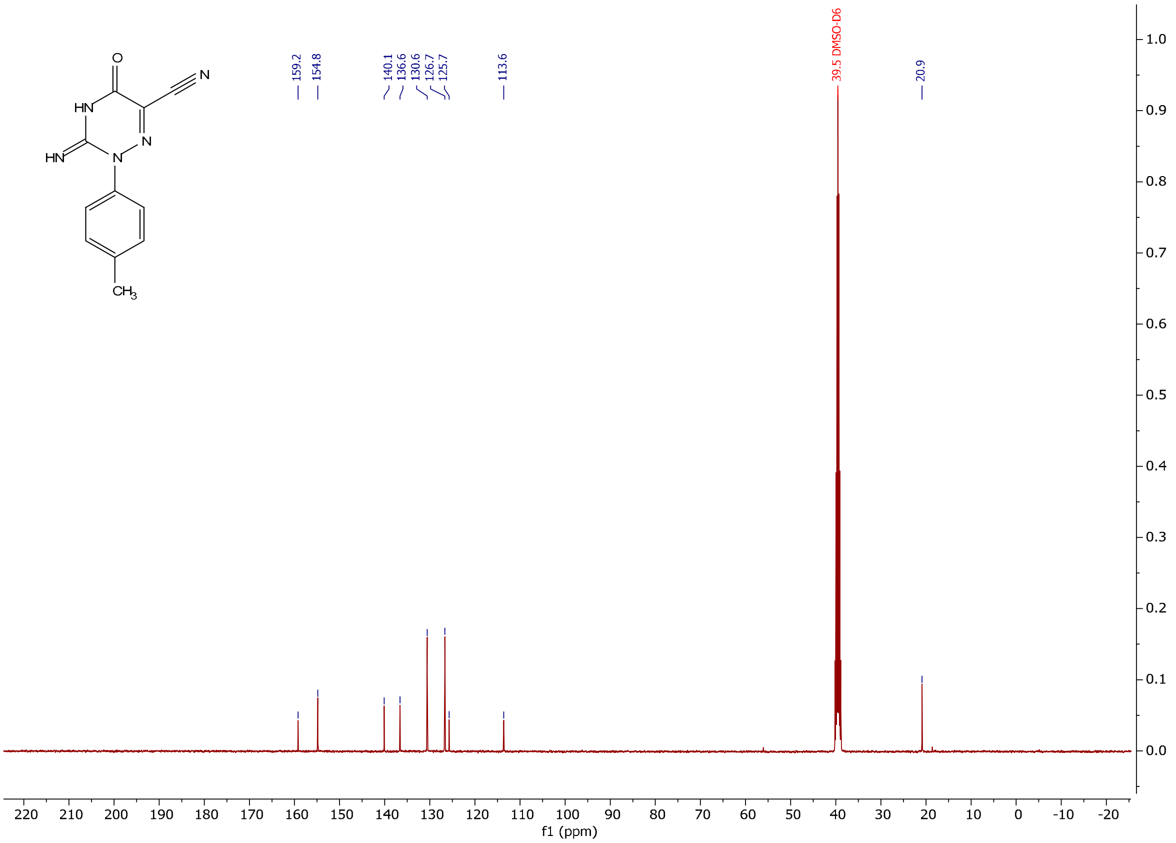

^{1}H spectrum of 4c:

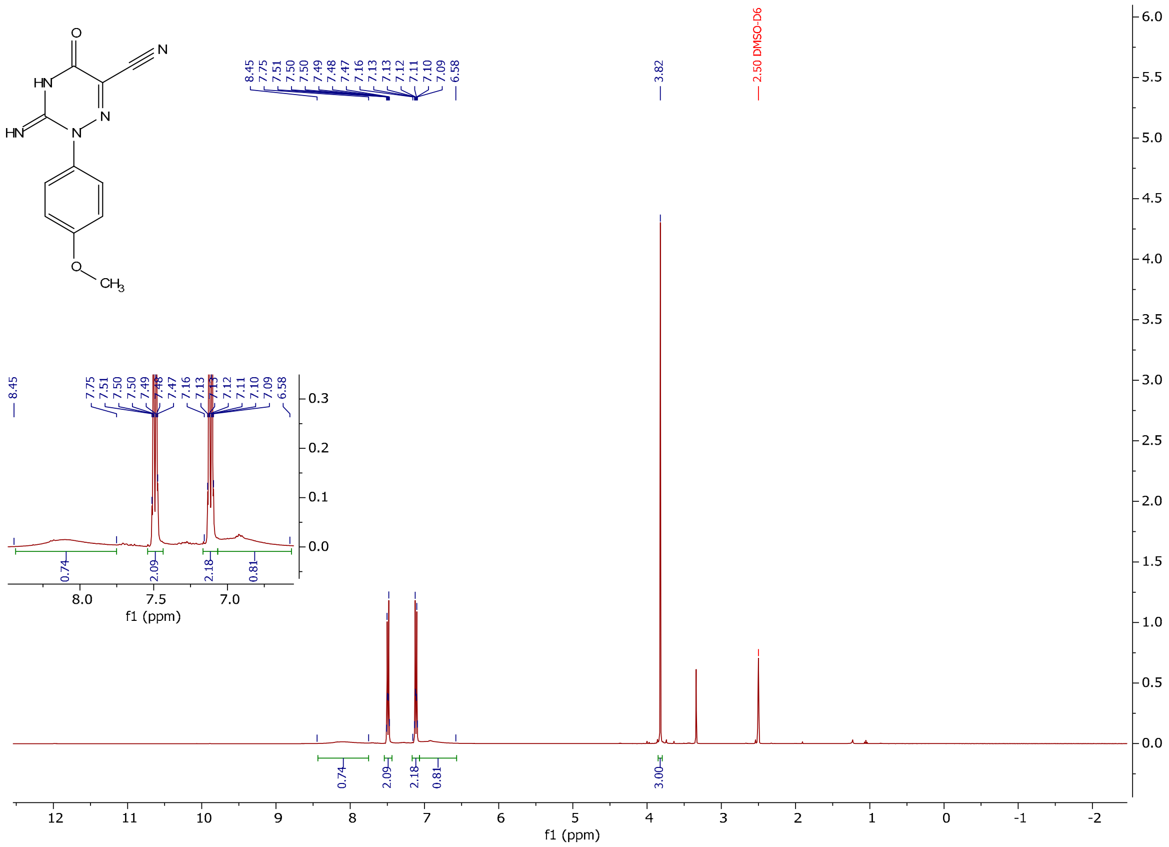

^{13}C spectrum of 4c:

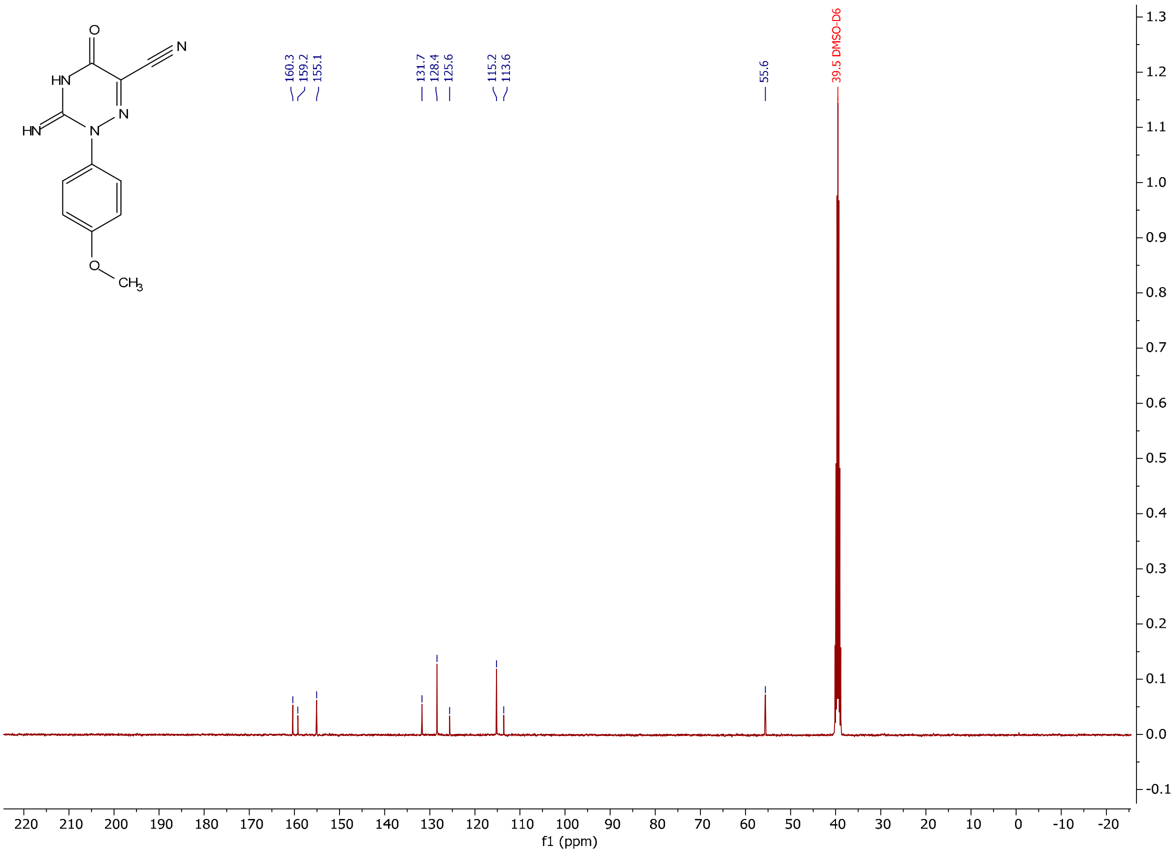

^{1}H spectrum of 4d:

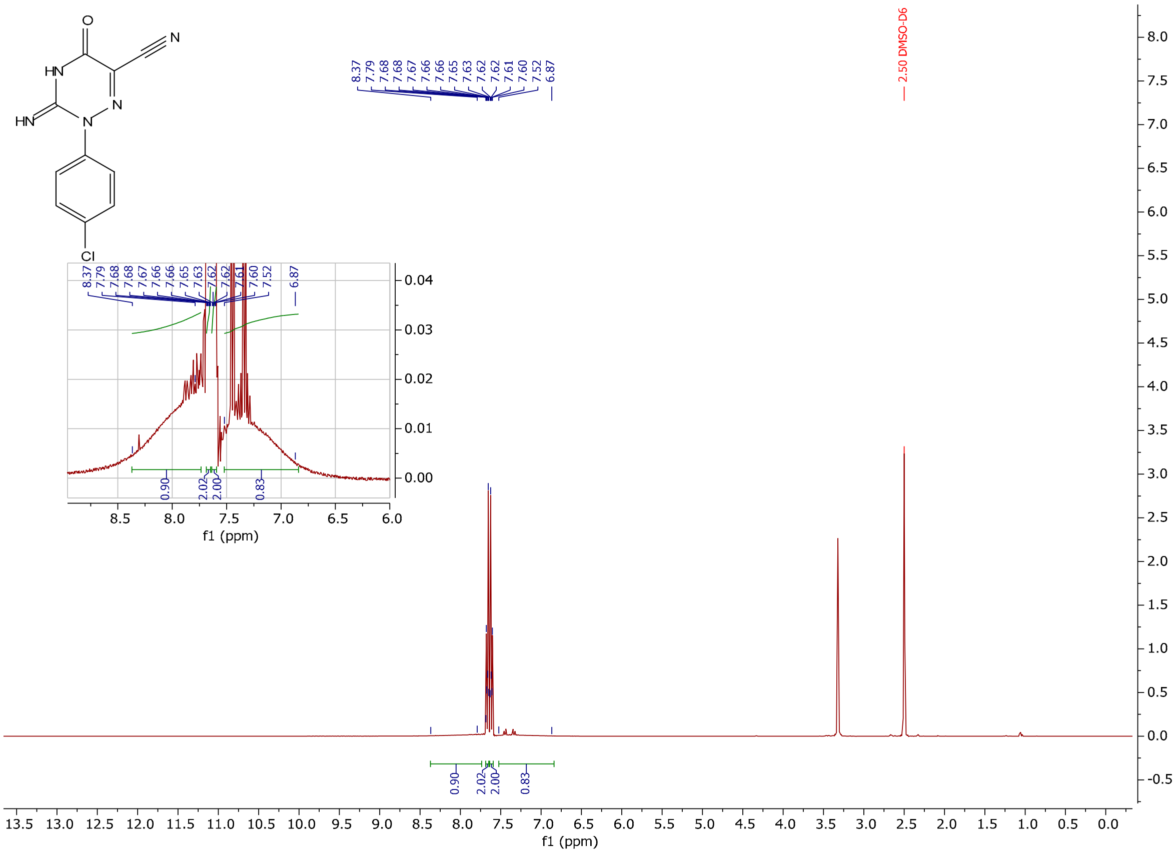

^{13}C spectrum of 4d:

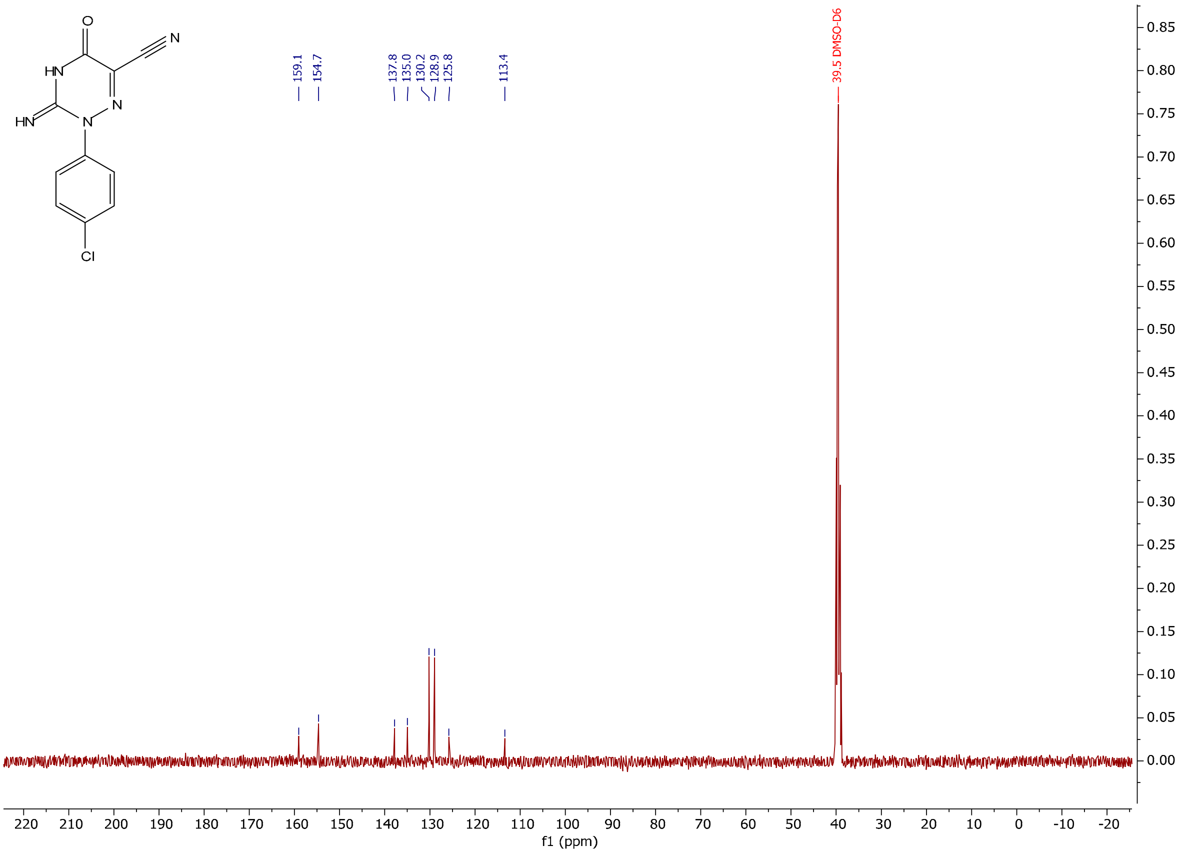

^{1}H spectrum of 4f:

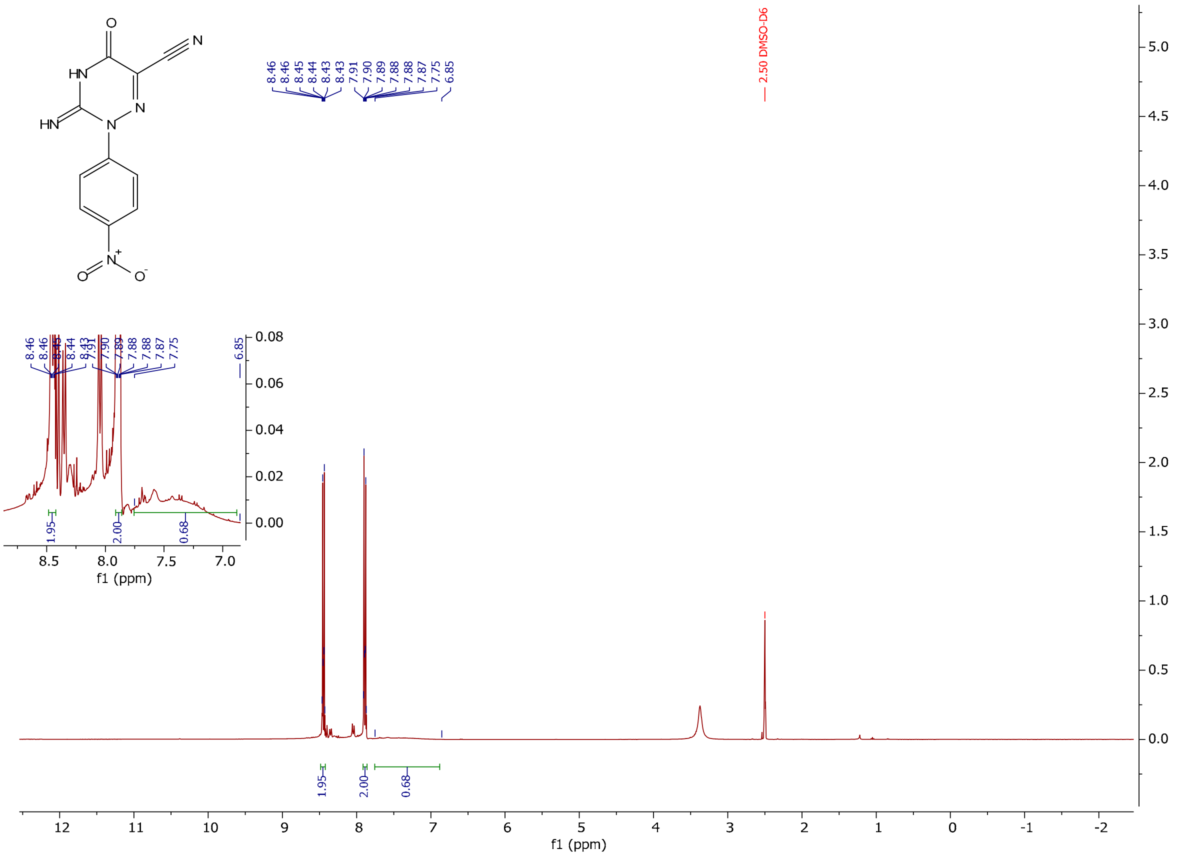

^{13}C spectrum of 4f:

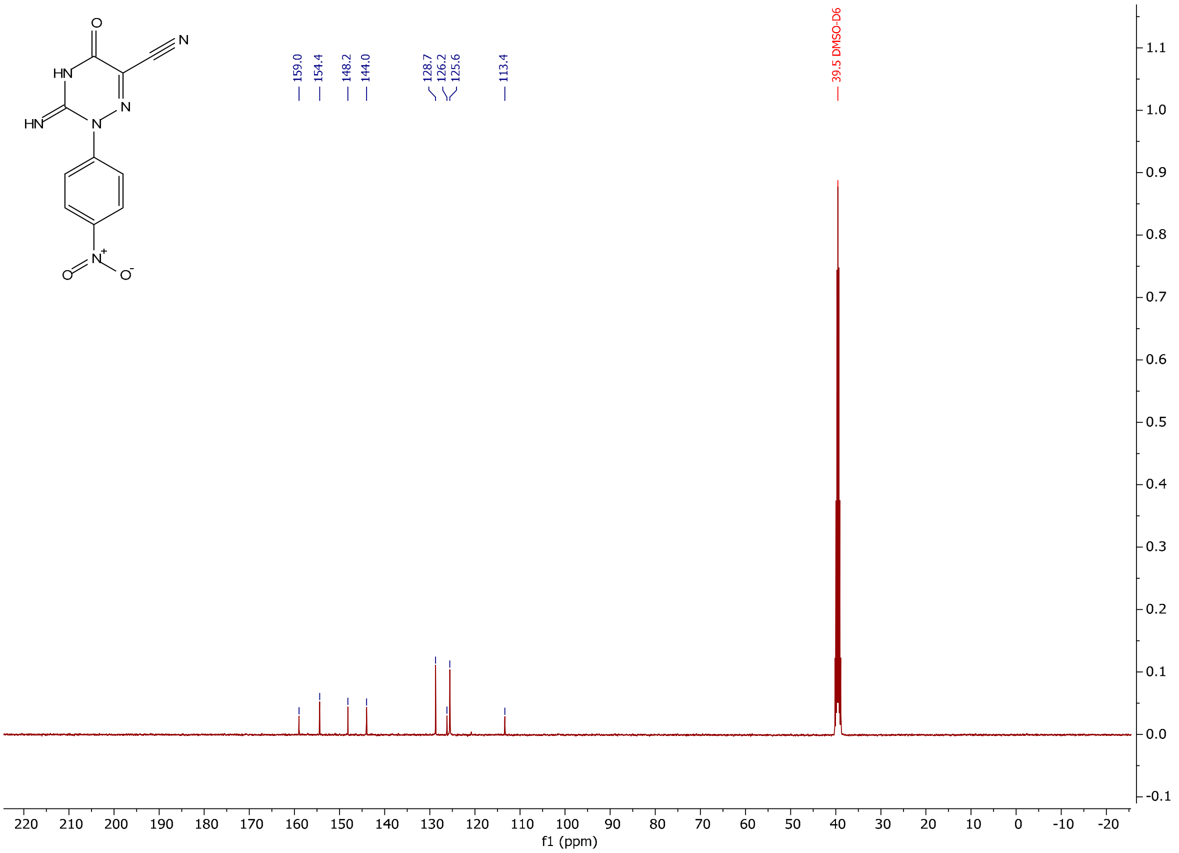

^{1}H spectrum of 5:

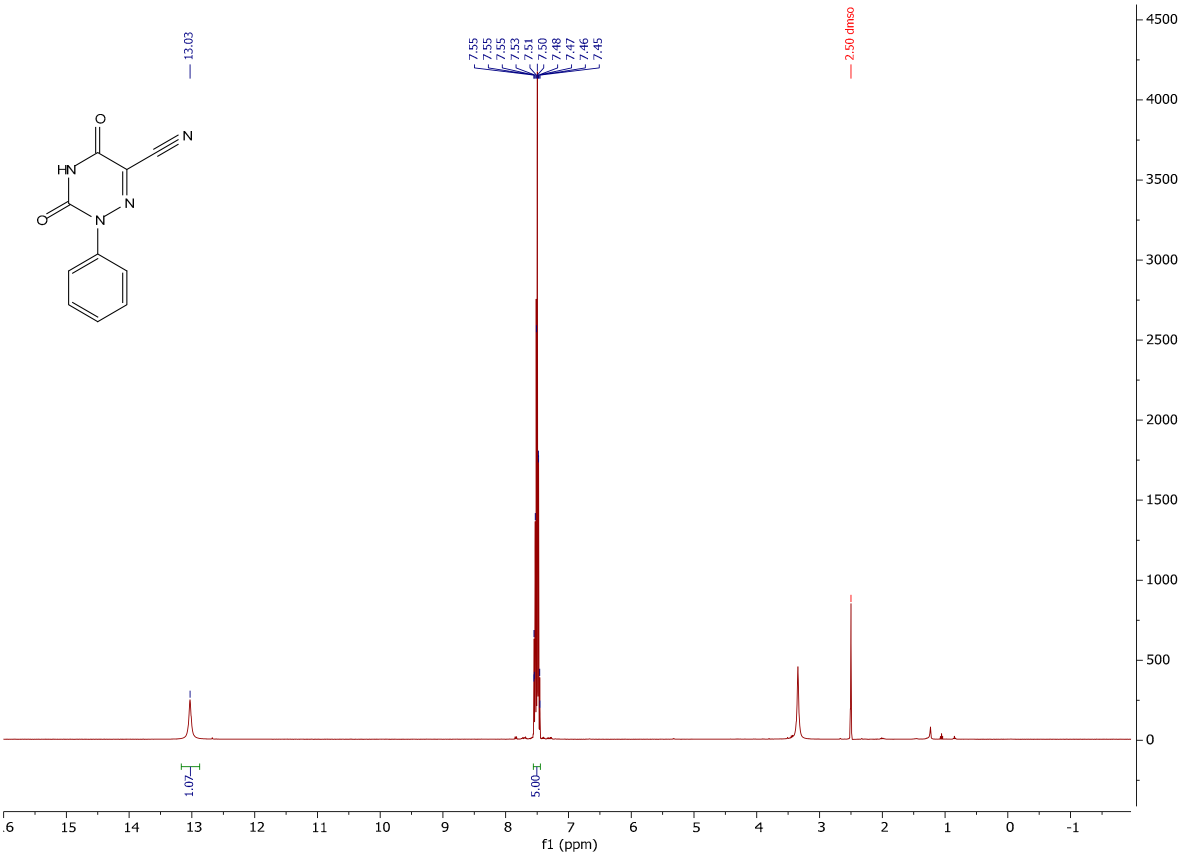

^{13}C spectrum of 5:

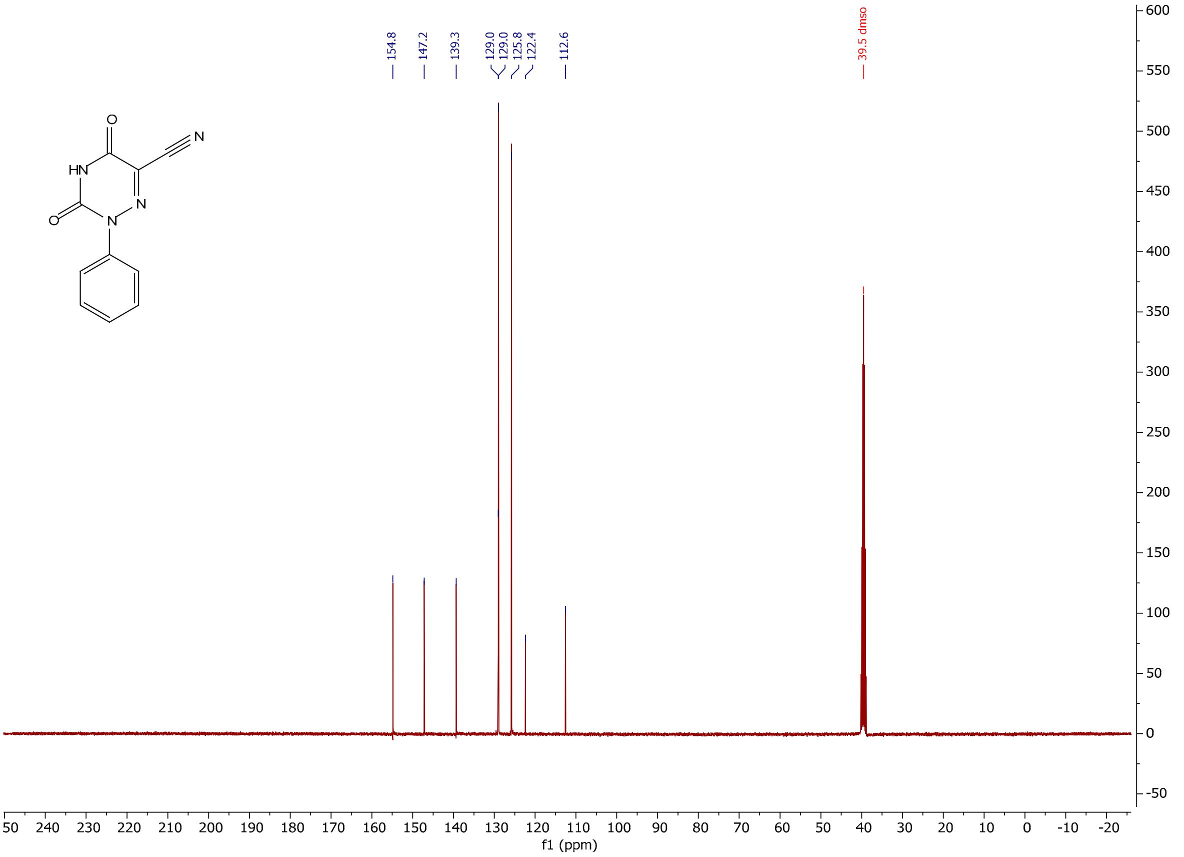

^{1}H spectrum of 6:

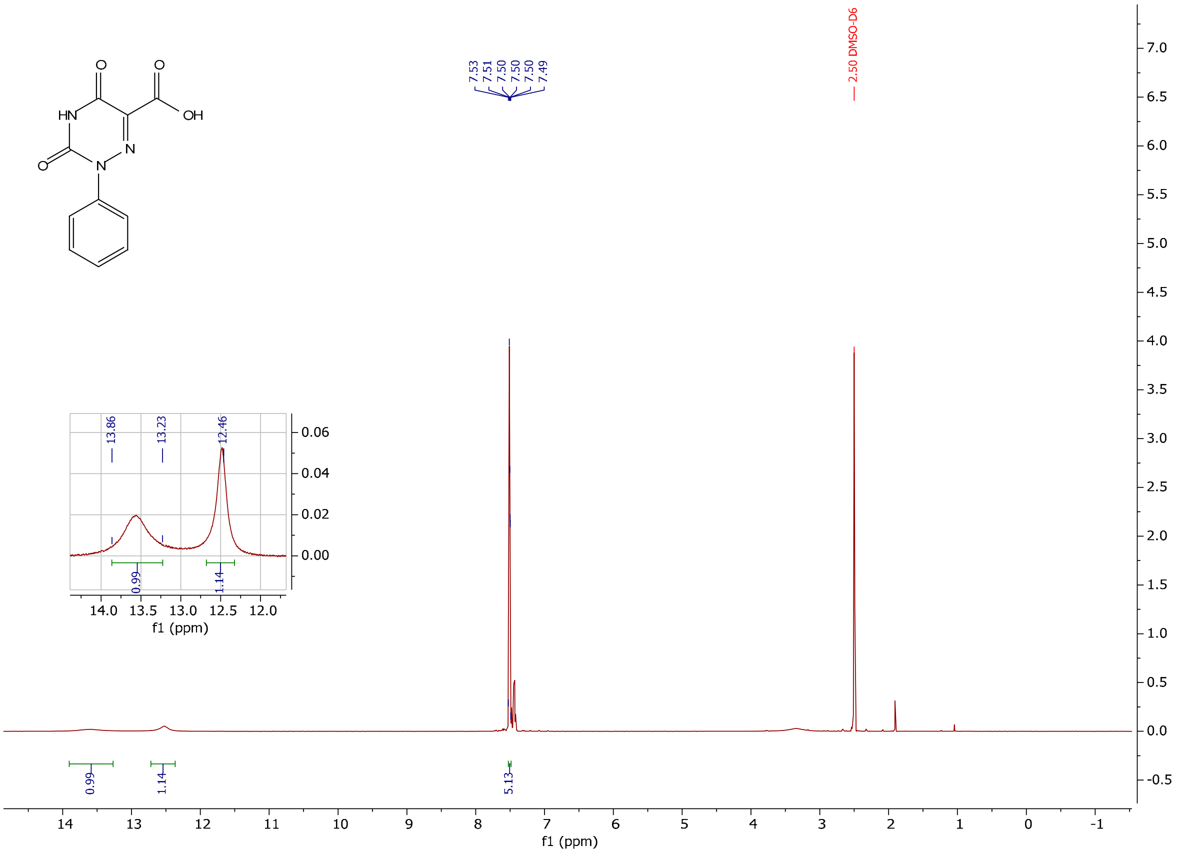

^{13}C spectrum of 6:

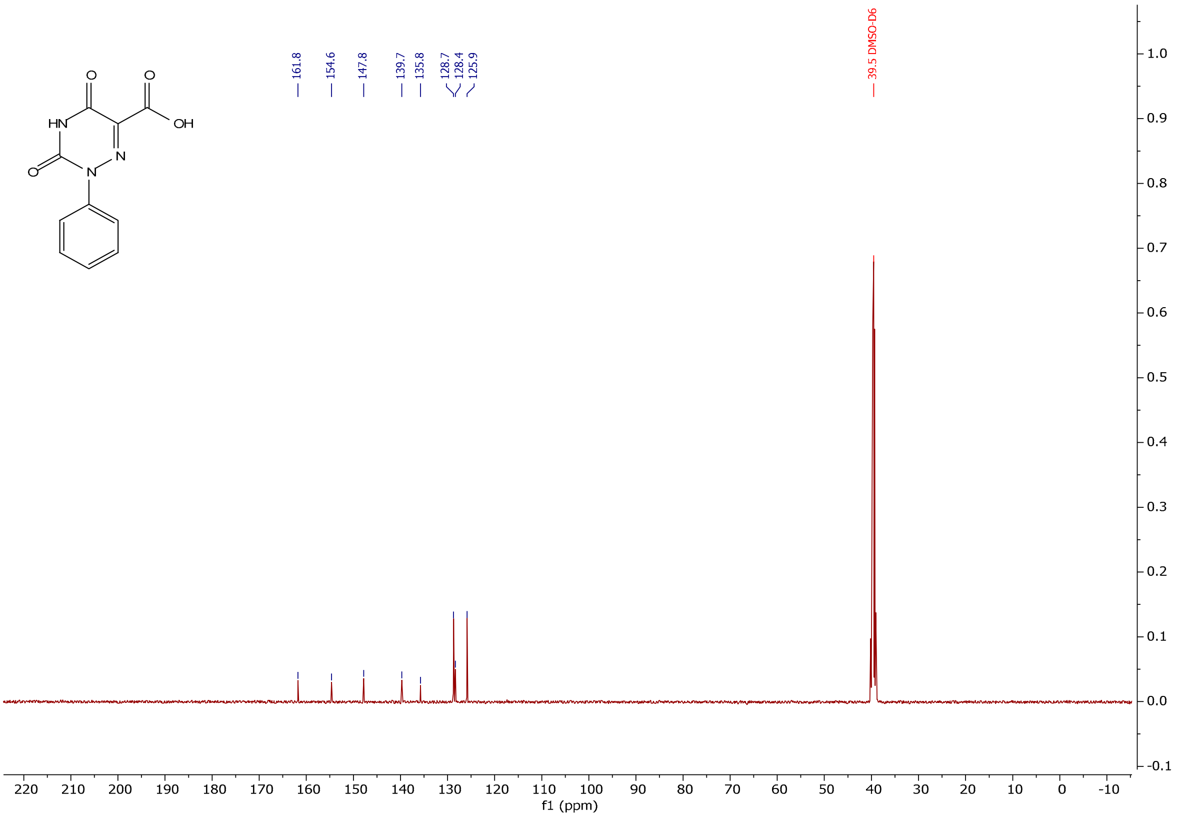

^{1}H spectrum of 8:

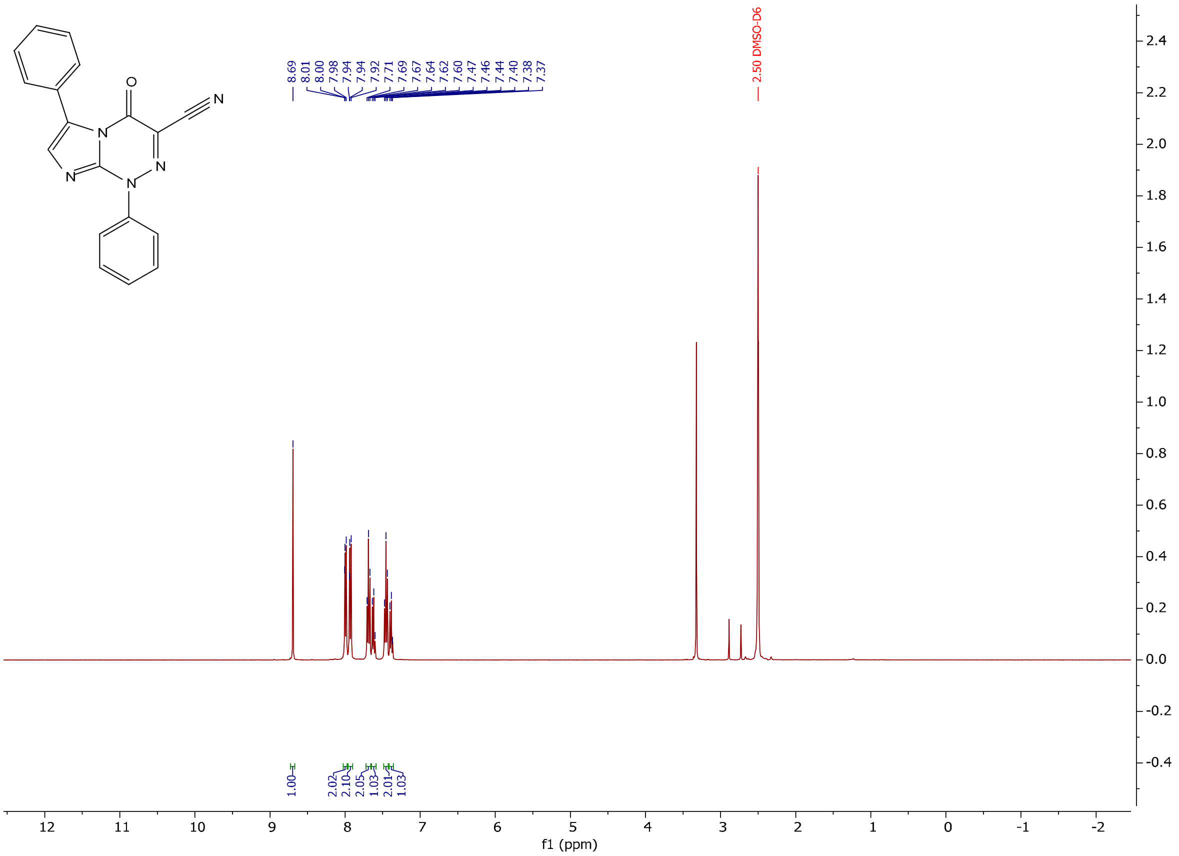

^{13}C spectrum of 8:

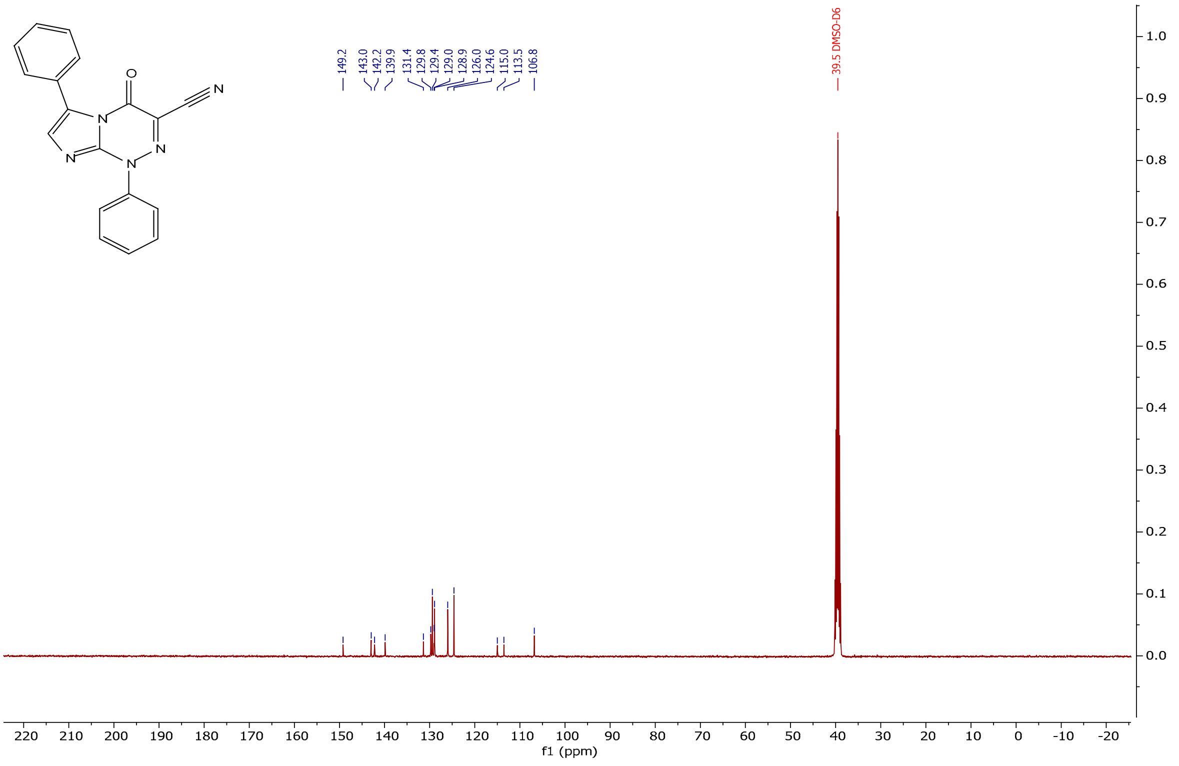

^{1}H spectrum of 9:

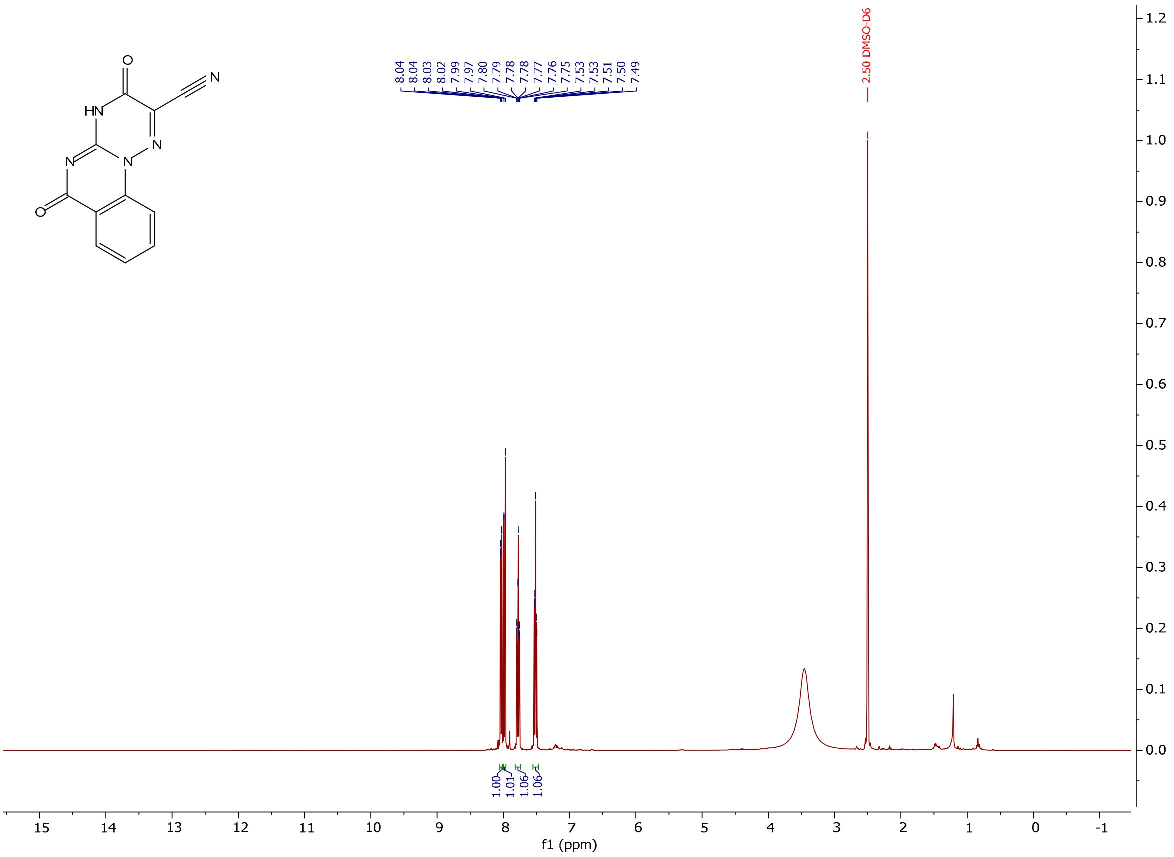

^{13}C spectrum of 9:

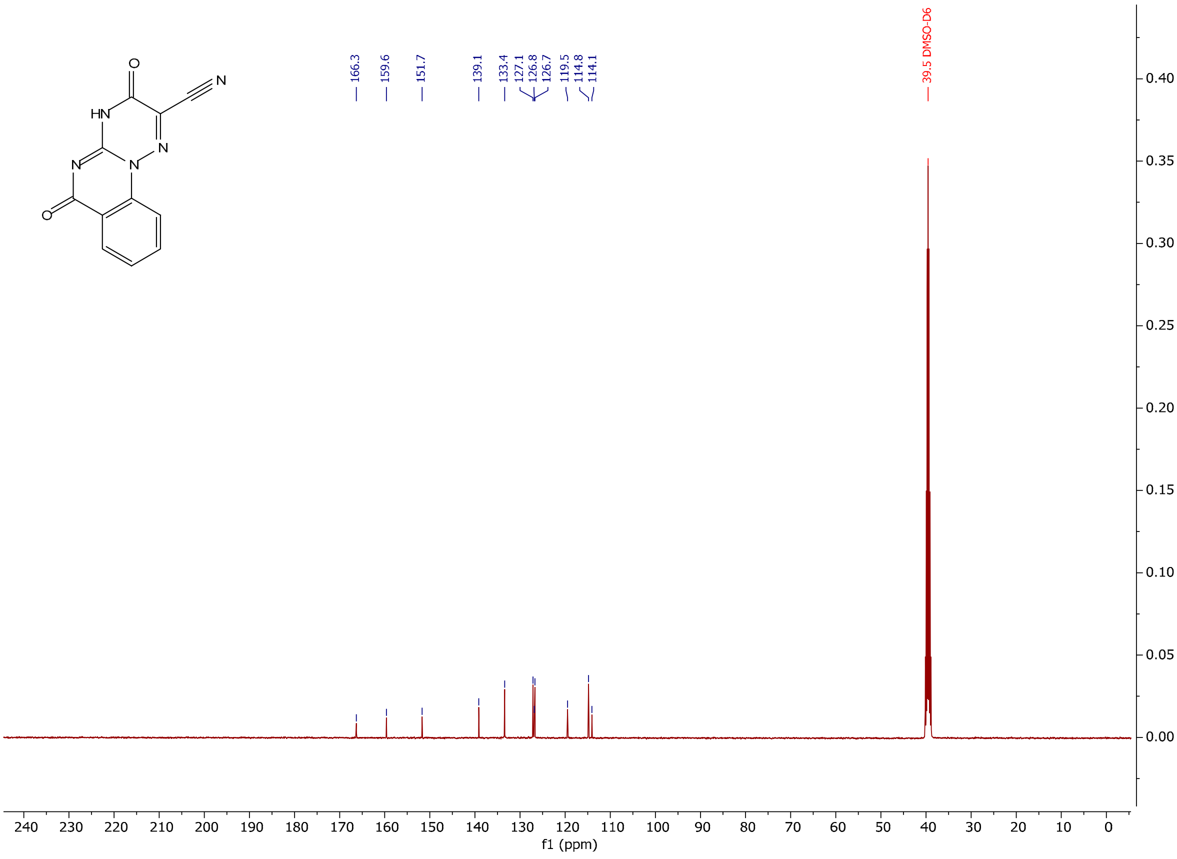

^{1}H spectrum of 10:

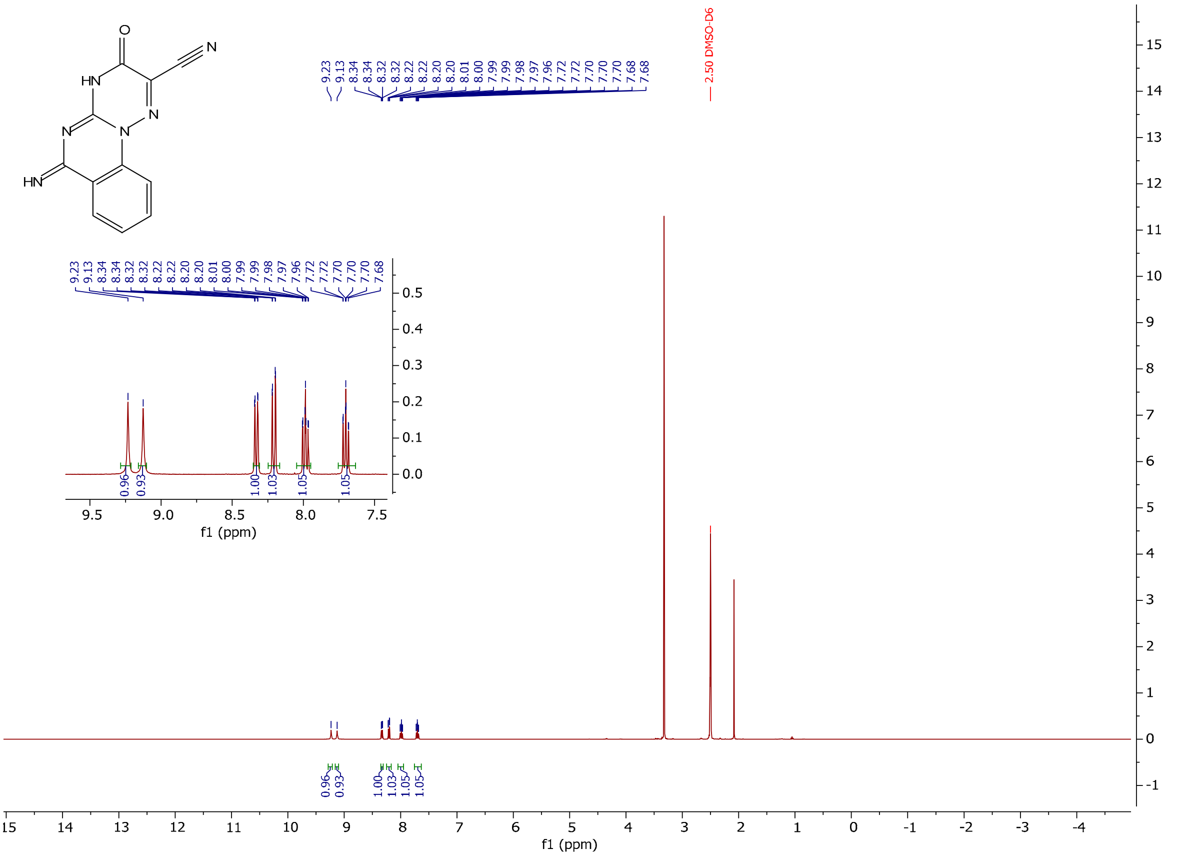

^{13}C spectrum of 10:

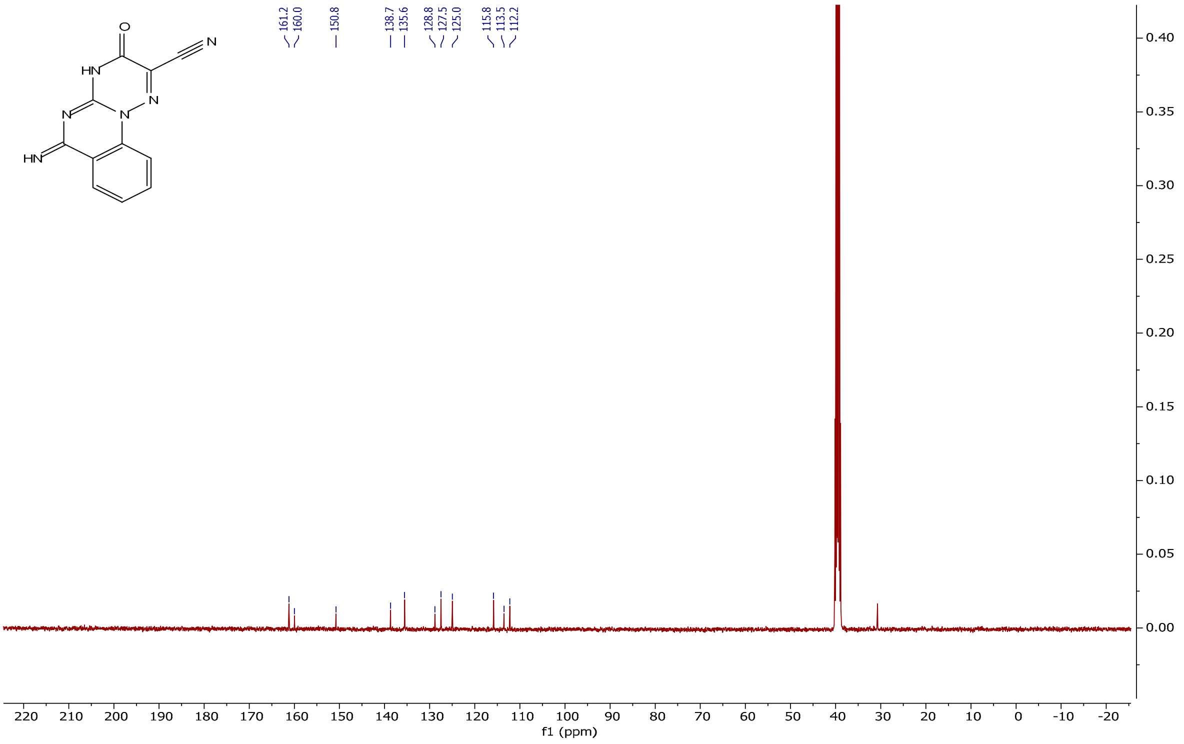

^{1}H spectrum of 11:

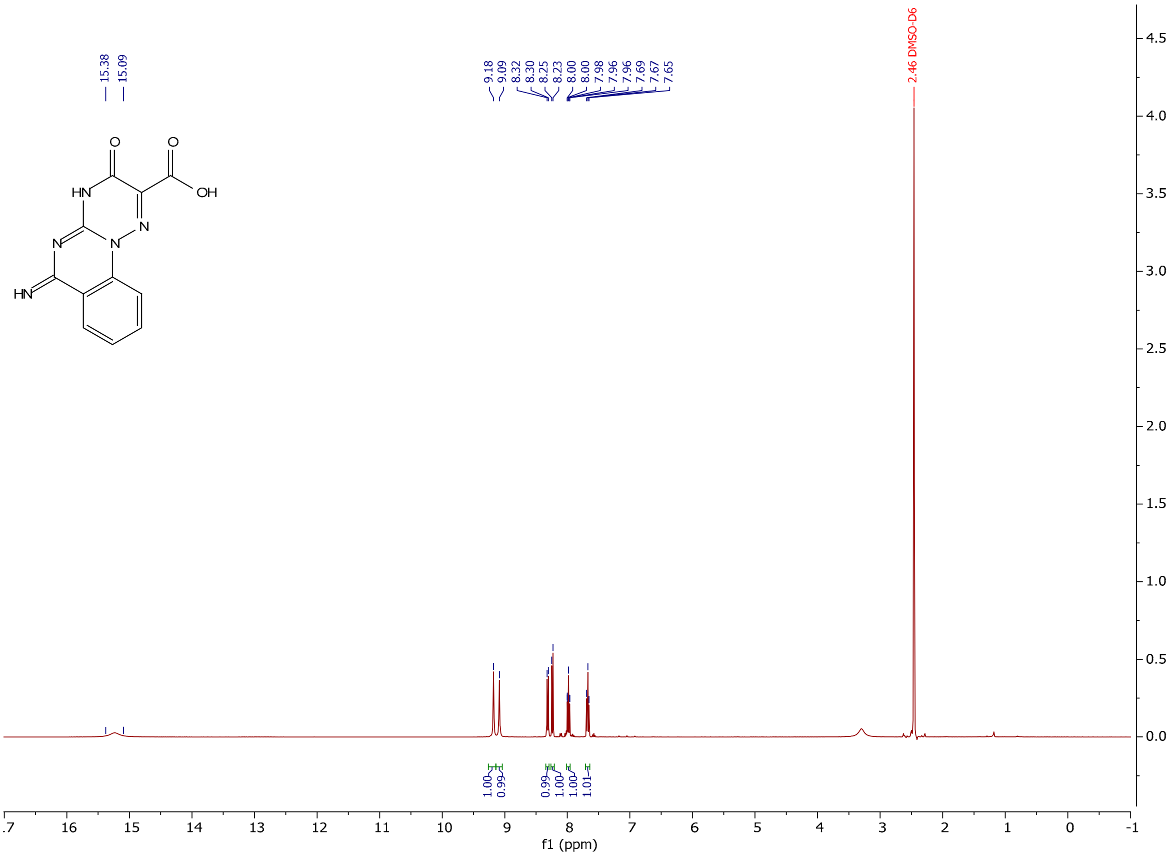

^{13}C spectrum of 11:

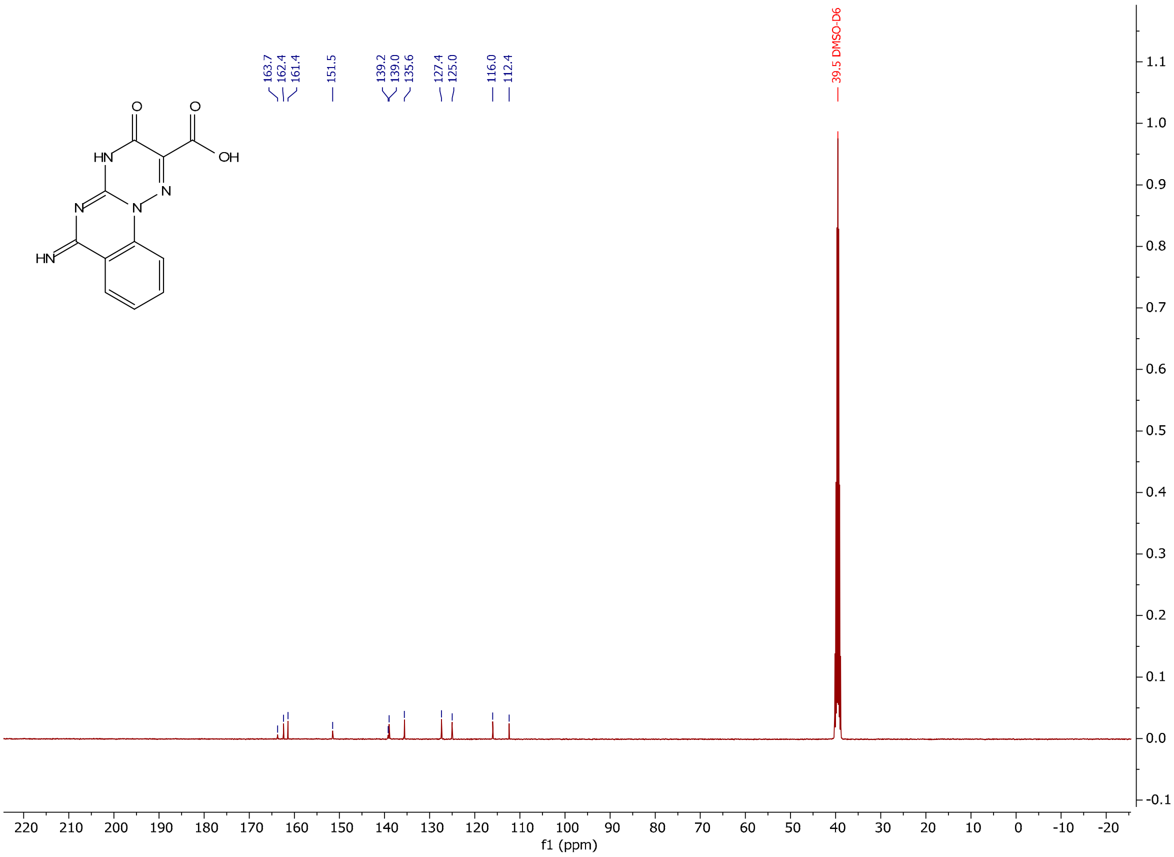

^{1}H spectrum of 12:

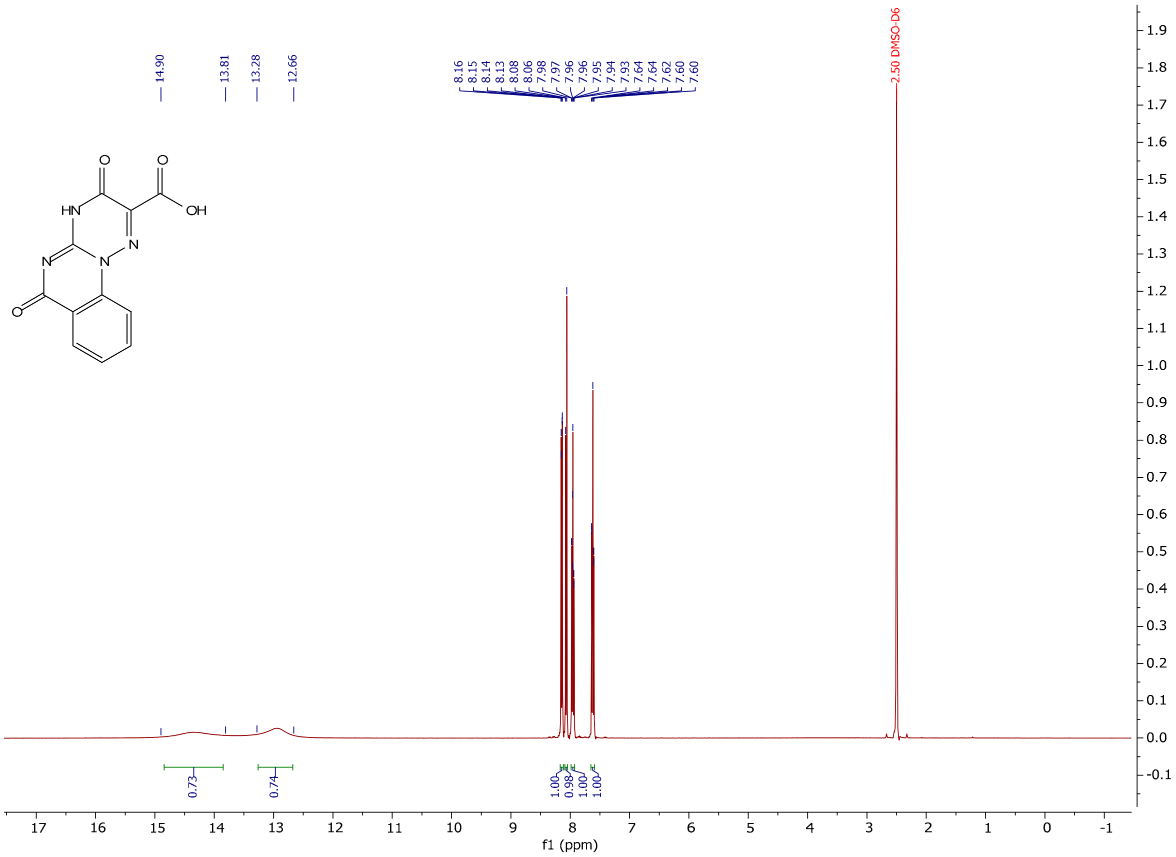

^{13}C spectrum of 12:

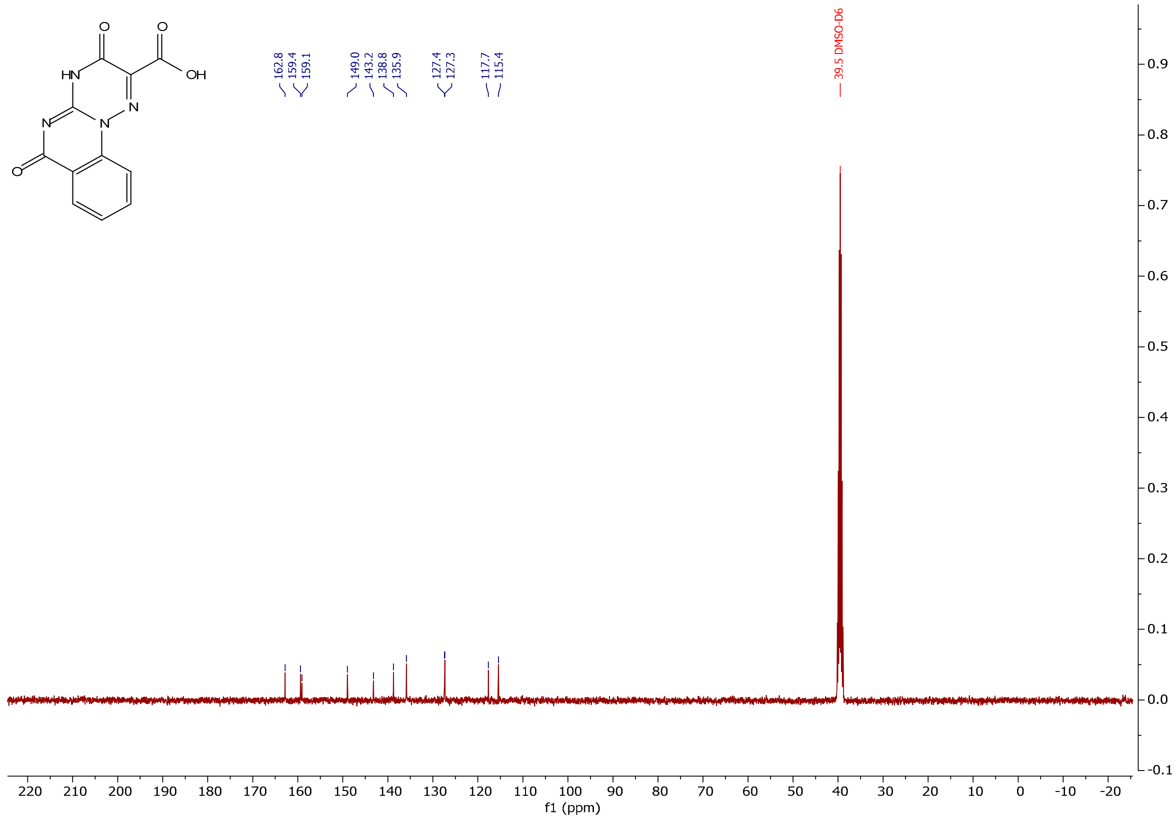

^{1}H spectrum of 13g in $CDCl_3$:

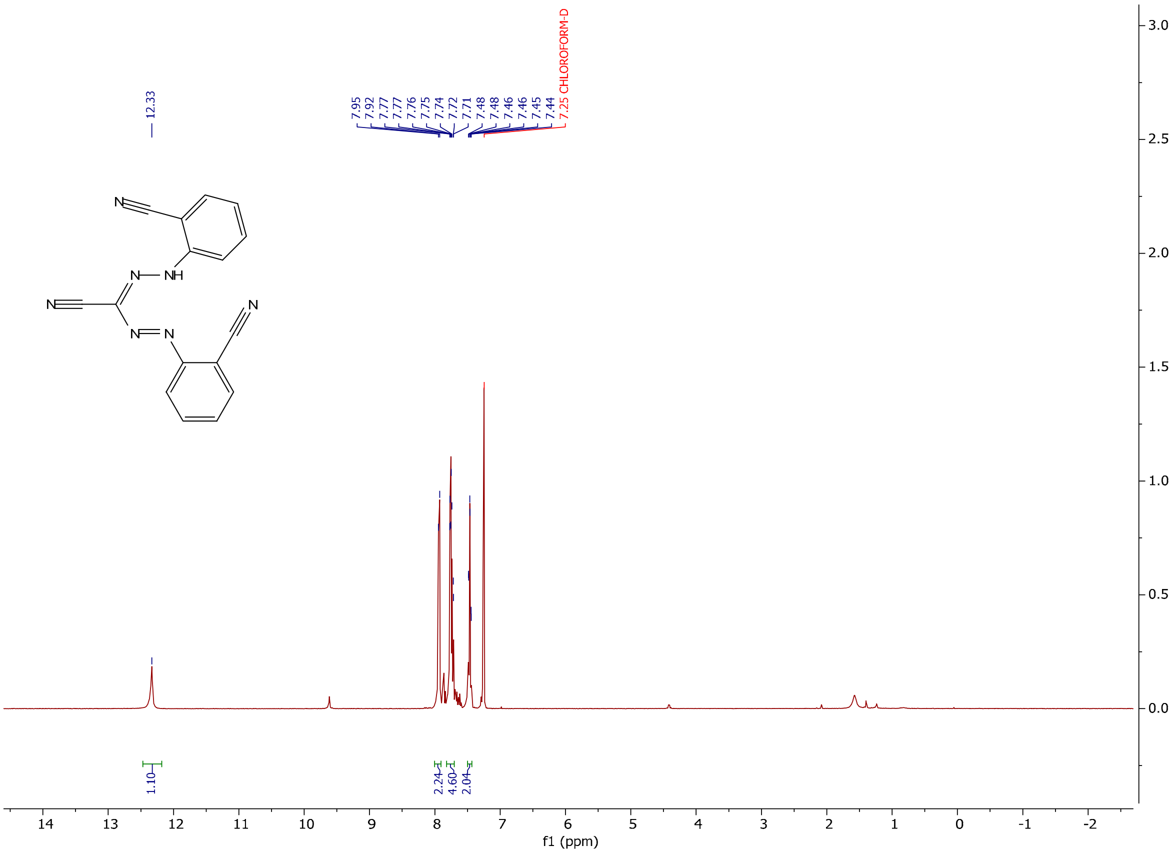

^{13}C spectrum of 13g in $CDCl_3$:

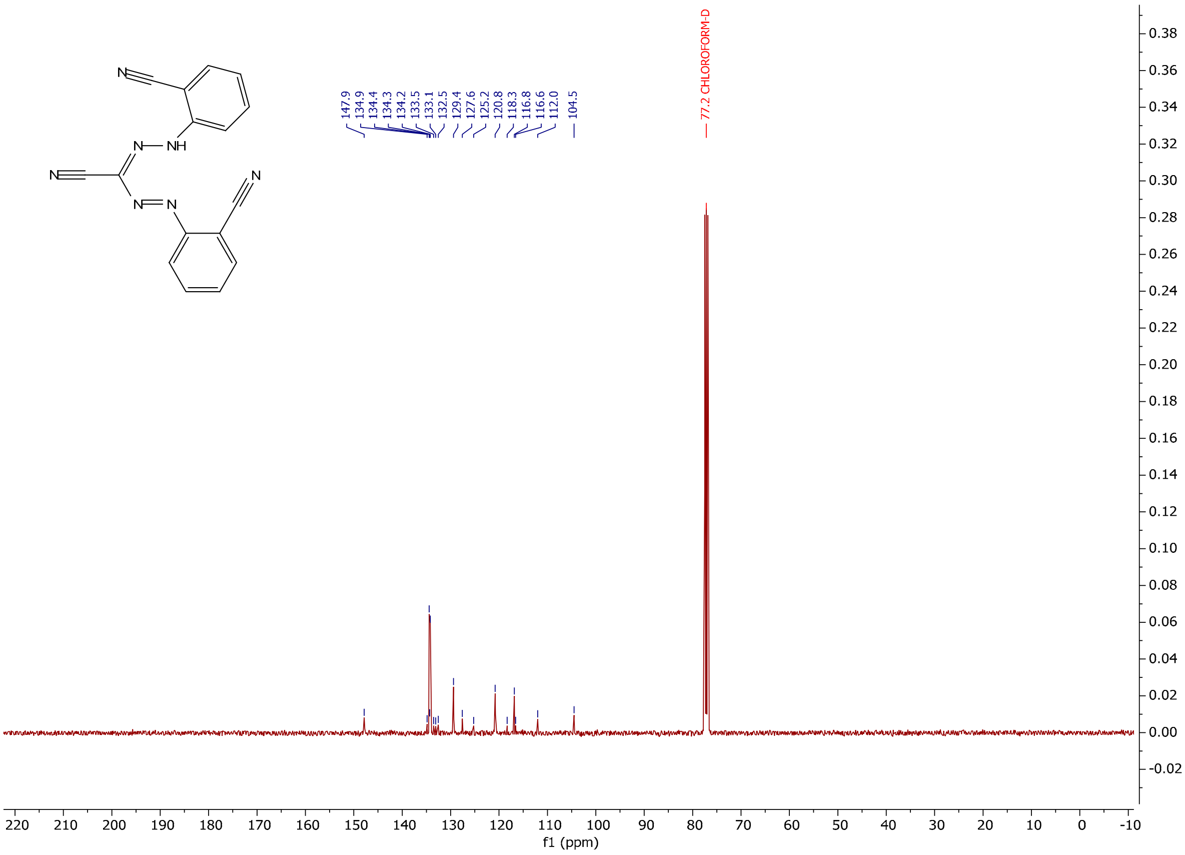

^{1}H spectrum of 13g in DMSO:

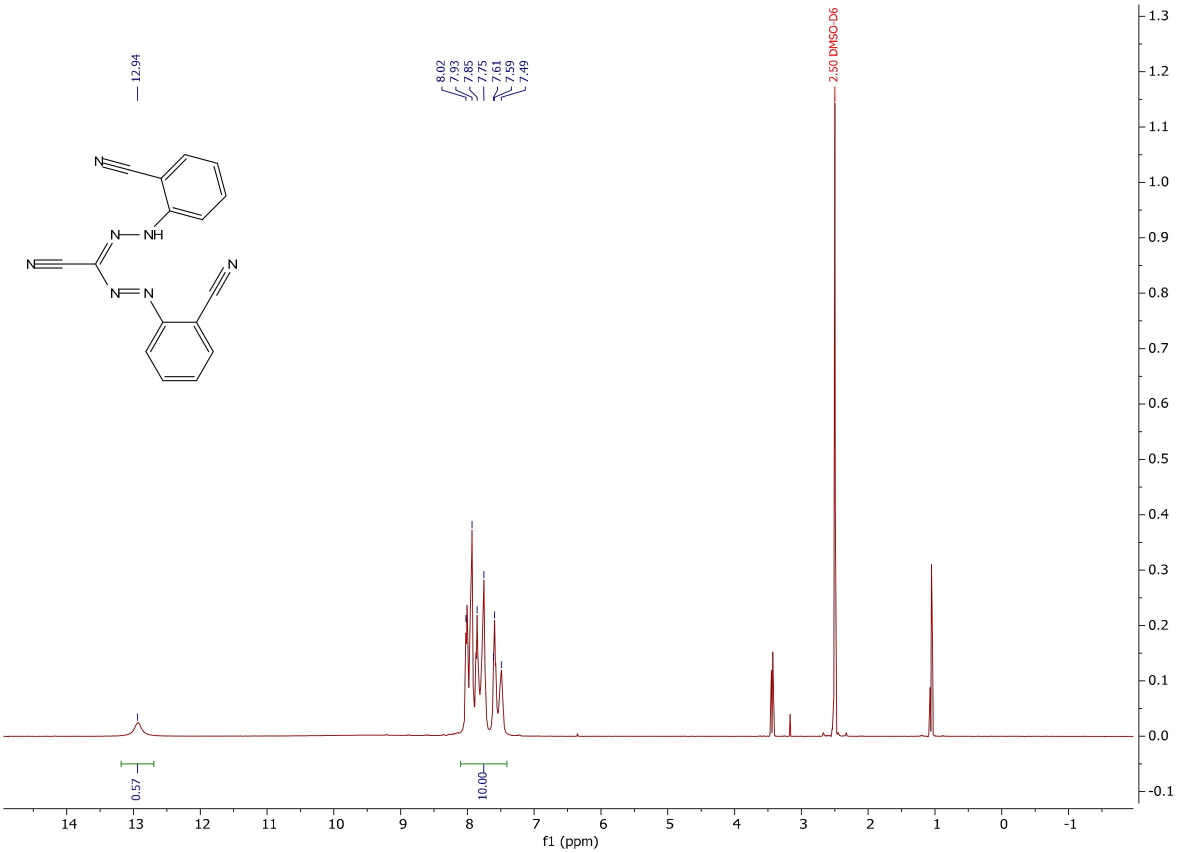

^{13}C spectrum of 13g in DMSO:

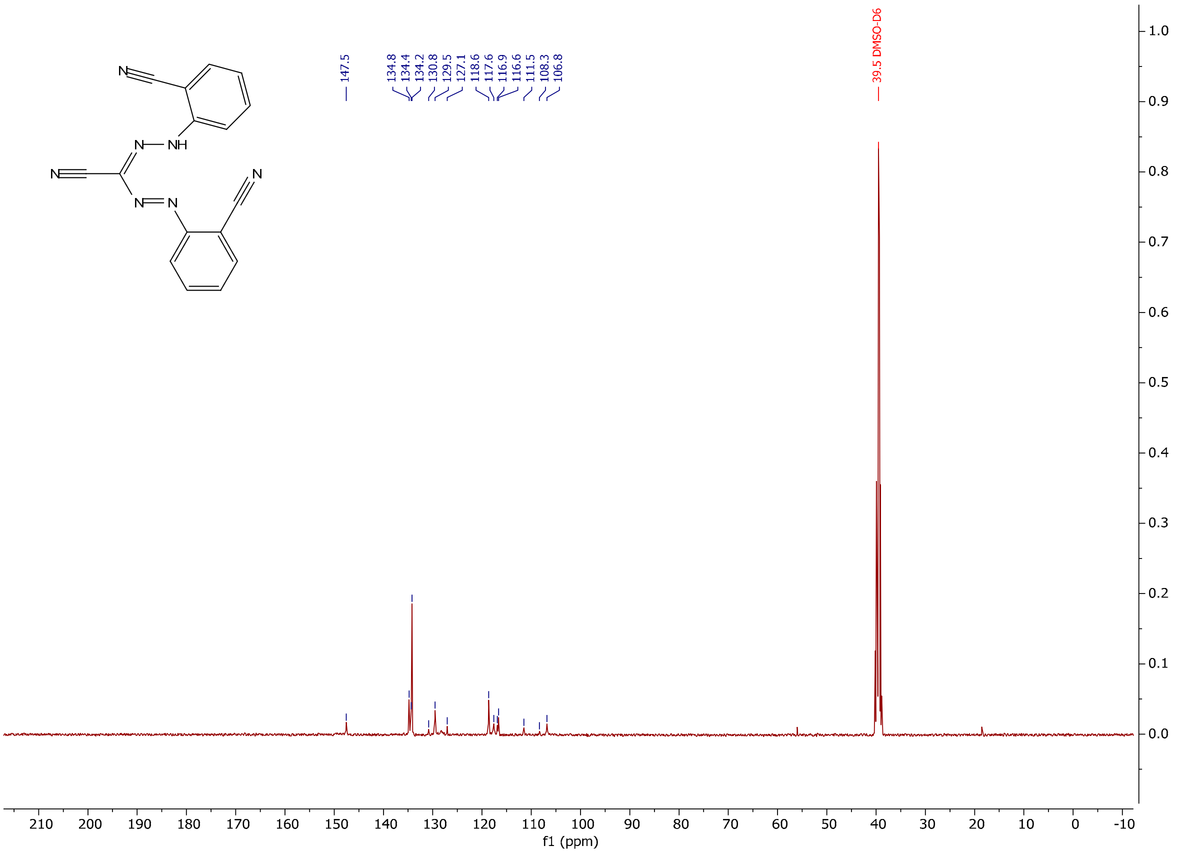

IR spectra of compounds 3g and 10

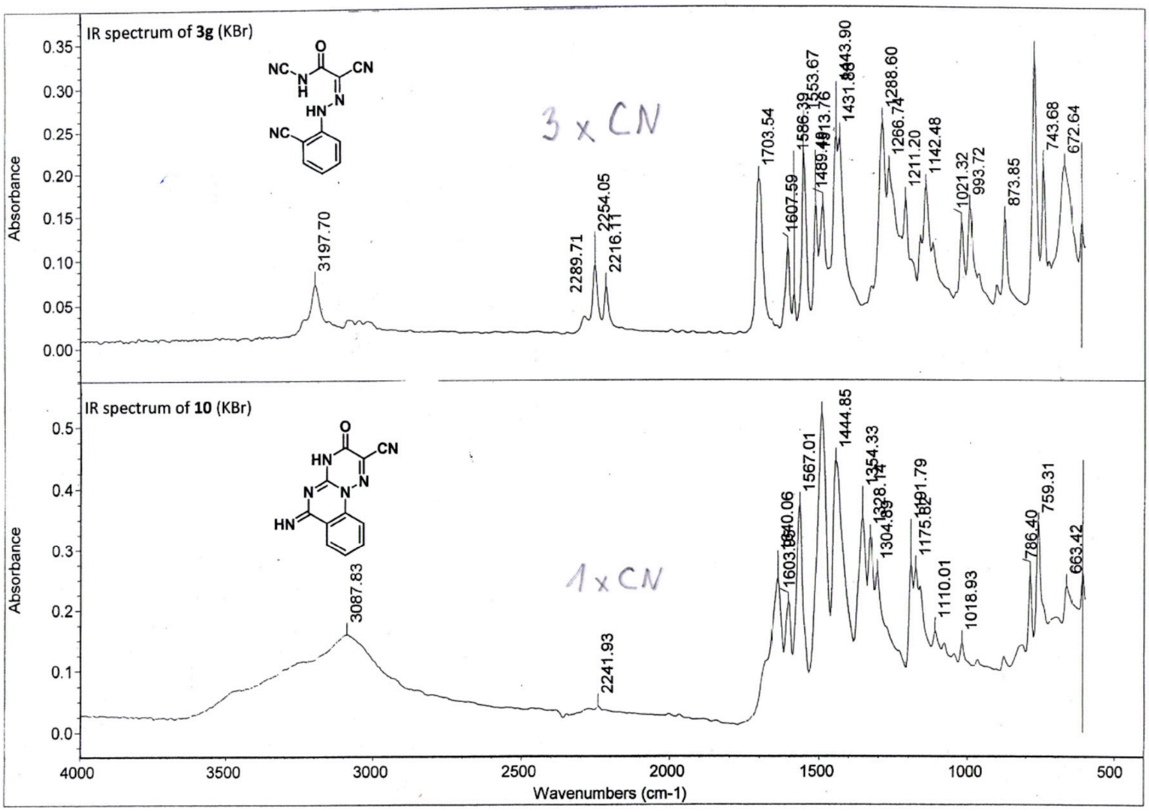

Supplement: Supplementary file 1 [file molecules-24-03558-s001.pdf]
